# Interstitial Fluid Flow and Drug Delivery in Vascularized Tumors: A Computational Model

**DOI:** 10.1371/journal.pone.0070395

**Published:** 2013-08-05

**Authors:** Michael Welter, Heiko Rieger

**Affiliations:** Theoretical Physics, Saarland University, Saarbrücken, Germany; University of Arizona, United States of America

## Abstract

Interstitial fluid is a solution that bathes and surrounds the human cells and provides them with nutrients and a way of waste removal. It is generally believed that elevated tumor interstitial fluid pressure (IFP) is partly responsible for the poor penetration and distribution of therapeutic agents in solid tumors, but the complex interplay of extravasation, permeabilities, vascular heterogeneities and diffusive and convective drug transport remains poorly understood. Here we consider–with the help of a theoretical model–the tumor IFP, interstitial fluid flow (IFF) and its impact upon drug delivery within tumor depending on biophysical determinants such as vessel network morphology, permeabilities and diffusive vs. convective transport. We developed a vascular tumor growth model, including vessel co-option, regression, and angiogenesis, that we extend here by the interstitium (represented by a porous medium obeying Darcy's law) and sources (vessels) and sinks (lymphatics) for IFF. With it we compute the spatial variation of the IFP and IFF and determine its correlation with the vascular network morphology and physiological parameters like vessel wall permeability, tissue conductivity, distribution of lymphatics etc. We find that an increased vascular wall conductivity together with a reduction of lymph function leads to increased tumor IFP, but also that the latter does not necessarily imply a decreased extravasation rate: Generally the IF flow rate is positively correlated with the various conductivities in the system. The IFF field is then used to determine the drug distribution after an injection via a convection diffusion reaction equation for intra- and extracellular concentrations with parameters guided by experimental data for the drug Doxorubicin. We observe that the interplay of convective and diffusive drug transport can lead to quite unexpected effects in the presence of a heterogeneous, compartmentalized vasculature. Finally we discuss various strategies to increase drug exposure time of tumor cells.

## Introduction

Cancer is a complex disease which involves phenomena across different scales from the molecular genetic level to the tissue as a whole. Cancerous cells of solid tumors have undergone mutations all of which combined lead to cancer [1]. These involve a dysfunctional control of proliferation, the ability to survive under low nutrient conditions and the stimulation of increased vascularization through angiogenesis [2]. This leads to an advantage in the competition over space and nutrients whereby cancer cells are also able to evade the immune systems which would otherwise kill malfunctioning cells. Solid tumors grow as compact masses. In order to grow larger than a few millimeters they must acquire additional nutrient supply through a blood vessel network. In response to inadequate supply cells produce signaling substances called growth factors which diffuse through the tissue and stimulate sprouting of new blood vessels from preexisting host vessels (angiogenesis). In tumors this angiogenic activity is located within a few hundred micrometers from the tumor rim. Fueling further growth, the resulting neovasculature is progressively co-opted together with the original blood vessels by the expanding tumor mass while also pushing the neovascularization zone further into normal tissue. Chemical signaling by the tumor is however abnormal, leading to chaotic non-hierarchical vascular organization. Behind the invasive edge, angiogenic activity ceases. Further proliferation of endothelial cells instead leads to circumferential growth and dilated tumor vessels. Also vessel walls degenerate via detachment of structural support cells like pericytes and smooth muscle cells. In conjunction with decreased blood flow rates vessels become prone to collapse leading to large unvascularized regions. Some surviving vessels thread the tumor, distal to which the tumor tissue becomes necrotic due to the lack of nutrients. As a whole such a typical tumor vasculature is characterized by tortuous vessels, chaotic connectivity and heterogeneous distribution as well as a compartmentalization into a zone with high micro-vascular density (MVD) near the invasive edge and a rapid density drop towards the center.

The interstitial fluid (IF), which is a solution that bathes and surrounds the human cells and originates from blood plasma extravasating from capillaries through pores and intercellular clefts in the vessel wall, plays an important role in the development and treatment of tumors. Due to degenerate walls many tumor vessels are leaky leading to a stronger coupling of the interstitial fluid pressure (IFP) with the blood pressure. This leads to an interstitial hypertension which can be elevated up to the blood pressure. The resulting decreased pressure difference across vessel walls is believed pose a barrier to drug delivery due to decreased convective trans-vascular transport or even back-flow [3]–[8]. Moreover functional lymphatics which would normally drain the superfluous fluid are absent in most tumors aggravating the IFP increase [2]. As a result the IFP profile assumes a plateau in the center of the tumor and drops off rapidly across the boundary. This gradient drives a strong outward directed convective flow at around 0.1 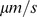. Signaling chemicals or tumor cells can therewith be transported into normal tissue or into lymphatics, promoting invasive behavior and metastatization [8], [9]. Indeed high IFP is associated with a negative prognosis. High IFP also has negative implications for chemotherapeutic treatment. Through the outward convection, drug may be removed from the peripheral regions.

Mathematical modeling of interstitial fluid flow and delivery was first approached in a radially symmetric geometry with homogeneously distributed source and sink terms using a porous media model for the interstitial flow velocity [3]. This predicted in agreement with experimental results an elevated IFP profile and the corresponding velocity profile. Convective (drug) extravasation was virtually limited to a small peripheral region. Later, extensions were developed using explicit representations of the blood vessel network in two dimensions. In [10] individual vessels were arranged as rectangular grid and their blood pressure was coupled to the IFP at their lattice sites, including the effect of fluid loss through the vessel walls. A similar approach was taken in [11] but with a tumor vasculature which was generated from in-growth from two parent vessels. Except for [3] these studies did not consider drug transport. Recently, simulations of IF flow and drug transport were conducted based on imaging data from real tissues [12]. An analysis of biophysical parameters governing the distribution of the local drug concentration was performed in [13] primarily focusing on the effects of varying tissue permeabilities for diffusing drugs. The modeling incorporated a tumor vasculature, realistic tumor lesions and cellular uptake and binding. However convective transport was neglected. In [14] a model was introduced in which interstitial fluid interacts with a growing tumor, also incorporating a vascular network that evolves dynamically from an initial capillary grid. IFF and hence convective transport of macro-molecules depend crucially on the spatial distribution and strength of IF sources and sinks within the tumor, which are determined by the spatial arrangement of blood vessels together and their local blood pressure. Even when lymphatics are absent within the tumor, leaky vessels with low blood pressure represent also sinks for IFF inducing non-trivial flow patterns inside the tumor with unexpected effects for the convective transport of macro-molecules. It is clear that the predictive power of a computational model for IFF and drug delivery depends critically on the physiological relevance of the underlying model for the tumor vasculature. In the present paper we present for the first time a IFF and drug delivery study with a realistic, hierarchically organized arteriovenous initial vasculature, circumferential growth of tumor vessels and IF back flow into tumor vessels.

In earlier work we developed a mathematical model for vascularized tumor growth which involved an initial vasculature [15], [16] arranged as a grid and updating rules representing angiogenesis, dilation and collapse. More recently, it was extended with an arteriovenous initial vasculature where quite realistically few arteries and veins branch out in a tree-like manner down to the lowest level where they are connected by capillaries [17]–[19]. Using this framework, biophysical aspects of tumor blood flow and the spatial distribution of tumor blood vessel were analyzed but it did not involve the IF explicitly nor the presence of drugs.

In this paper we want to compute the interstitial fluid pressure and flow within a tumor and use this information to predict drug delivery within the tumor and its various dependencies on physiological parameters. These parameters include the blood vessel network morphology as opposed to simplified vasculature models, blood flow characteristics, blood pressure, permeabilities of the vessel walls, the interstitium and lymphatic walls, the mass of drug particles, i.e. the ratio of convection vs. diffusion. The tumor phenotype that we consider is a vascularized solid tumor for example a melanoma or glioma which grow in their natural environment in a human host with characteristic features as described above. Samples of such tumors were studied experimentally in [20], [21], [32].

One particular question is how far an elevated IFP is an obstacle to drug delivery. The general consensus is that elevated IFP reduces the convective flux through the vessel walls, due to the lowered pressure difference. However, our results indicate that this does not necessarily need to be true and that the relation between IFP and IFF is more complex, also involving vessel wall and tissue conductivity. Moreover our model predicts Peclet numbers (ratio of diffusion to convection) of the order of 1 in the tumor periphery. There the IF flow is largest and almost perpendicular to the boundary into normal tissue. Hence neither diffusion nor convection can be neglected. Finally our model also allows to study extreme cases like the delivery of very heavy drug particles, which are transported purely by convection. Their distribution is harder to predict than for a highly diffusive drug since it is dependent on long range transport along chaotic IF flow patterns which are eventually governed by vascular morphology.

This paper is organized as follows: Our mathematical model is defined in the next section. We first define the representation of the vessel network followed by remodeling rules during tumor growth and the procedure to construct the initial network. Then we define continuous parts, including the representation of tissue, modeling of tumor growth, IF flow, chemical concentration fields involved in tumor growth and finally drug transport. Finally a brief overview of our numerical implementation and a derivation of parameters are given. The subsequent results section comprises a discussion of a typical base case including a brief presentation of results obtained from tumor growth simulations and an in-depth analysis of IF flow and drug delivery. After that various other cases are analyzed before the paper is finally concluded.

## Model

**Figure 1:**
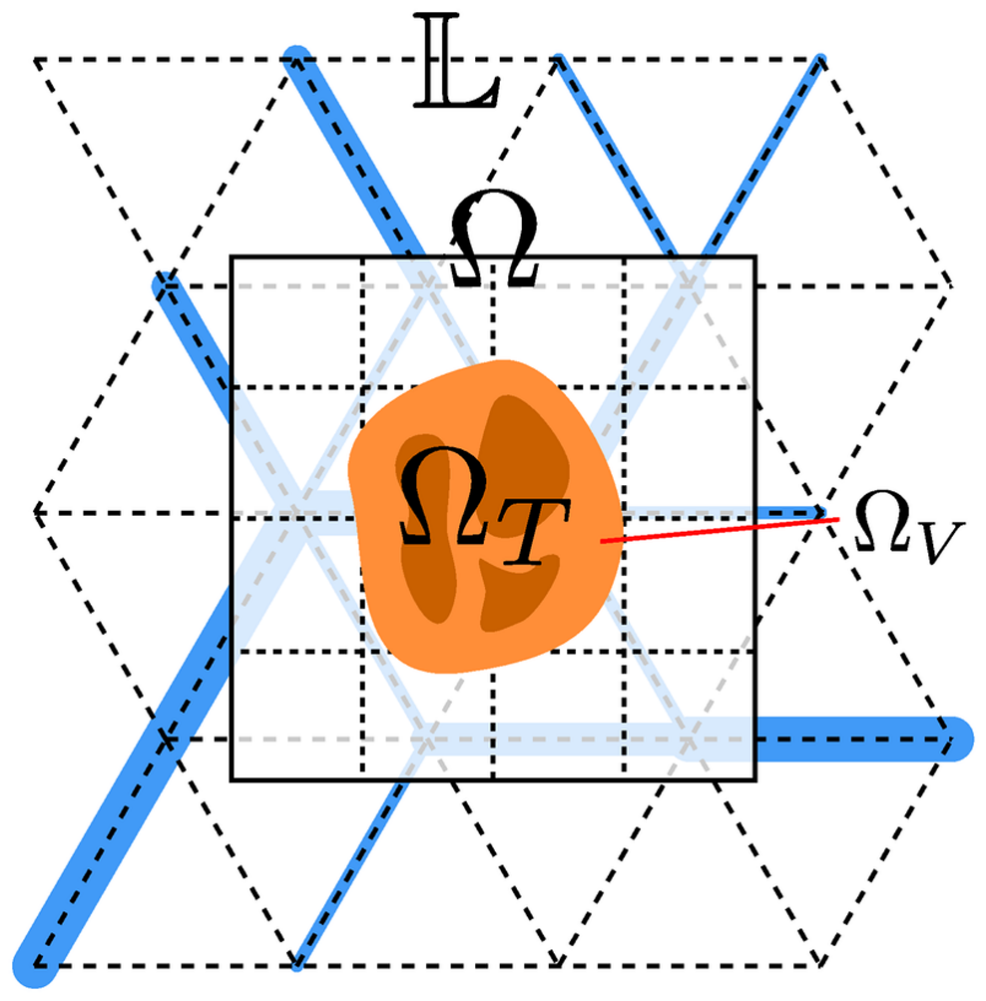
Illustration of the model components. 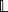
 denotes the lattice on which edges can be occupied with vessel segments. A few exemplary segments are shown as blue bars. 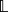 coexists with 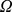 which denotes the region over that continuum equations are defined. The tumor region 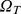 is indicated in yellow. The darker tone indicates necrotic regions. Viable regions, denoted 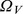 are brighter.

The model has been developed for simulations in three dimensions in Cartesian space. It is a hybrid model with discrete (vessels) and continuous parts (everything except vessels), see Figure 1 for an illustration. Continuous distributions are defined in the spatial domain 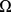 that we choose to be a cubic box. Discrete vessels are defined on a face centered cubic (FCC) lattice 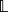, which has 60 branching angles between parent and child branches. Varying branching angles would require modeling the vessel network in continuous space, which is computationally much more demanding but would not change the large scale morphology of the resulting network. 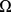 overlaps with 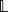 and both are centered at the origin. The lateral size of 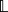 is 8 mm and that of 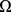 is 4.5 mm. The size of 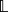 is chosen to be larger to reduce boundary effects. 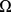 is initially filled with normal tissue and contains a small tumor nucleus located in its center.

We study IF flow and drug delivery for static tumor configurations. This means the tumor growth is simulated up to a specific time without explicit involvement of IF or modeling effects of drugs. Then IFP and IF flow are computed, and finally the spatiotemporal distribution of the drug concentration with the tumor frozen in time. A coupling would be interesting in the context of studying various therapeutic protocols, which we defer to forthcoming publications.

### Blood Vessel Network

Let 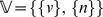 be the graph which formally describes vessels (edges) 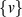 and junctions (nodes) 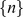. Vessels coincide with bonds from 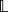, starting at one site of 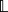 and ending at another. They can span multiple bonds but must be straight. Each vessel carries biophysical properties like radius or blood flow. We will introduce them as needed.

*Blood flow* is an essential part of our model. For vessels we compute the flow rate 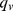 (volume through its cross-section per time) and shear stress on the vessel wall 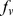 and the blood pressure 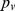 at the endpoints. The indices are dropped in the following. We assume ideal pipe flow within the vessels, obeying Hagen-Poisseuille's law
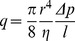


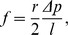
where 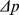 is the pressure difference between the vessel ends, 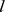 is the vessel length, 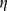 the blood viscosity and 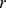 the vessel radius. 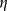 is composed of the blood plasma viscosity 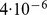 kPa s times the relative viscosity 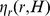 which is a function of the hematocrit 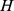 and the radius. For 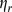 we use a formula based on in vivo experimental data [22]. For simplicity, we assume that 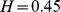, the average in the human body. Mass conservation at each node requires that the flow rates of attached vessels sum to zero: 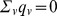 (Kirchhof's law). Together with appropriate boundary conditions a system of linear equations for the nodal pressures arises which is solved numerically. As boundary condition the pressure is fixed at the arterial and venous roots of the vascular trees. These boundary pressures are determined as function of the vessel radius, also guided by experimental data [23] (see Supplement S1 (1)). Note that we do not incorporate the extravasated fluid into the mass balance, which is justified since, as we will demonstrate in the results section, the amount of extravasated liquid is orders of magnitudes smaller than the total vascular blood flow. In the rest of the paper 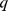 and 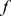 will denote the absolute value of the flow and shear force within a vessel - above they carried a sign.

*Blood vessel network remodeling*, the process in which the hierarchically structured initial network is reorganized by the growing tumor is defined by a set of stochastic and continuous processes which model angiogenesis, dilation, degeneration and collapse. They are implemented as updating rules which are applied consecutively in each time step. As a result, vessels are created, deleted or they change their properties. These rules are adopted straight forward from the 2d case [18] and presented here again for completeness.

Our time stepping scheme advances the vessel network in fixed steps of width 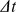. Assuming that the frequency of a stochastic event is determined by a rate parameter 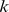 we approximate the probability for its occurrence in one time step as 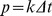. We chose 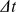 sufficiently small such that 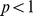.The time evolution of continuous processes described by differential equations of first order in time is handled by Euler's method with time step 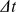. In the following we describe the individual vascular remodeling processes that are incorporated into our model, for an illustration of theses processes (see Supplement S1).

**Sprout initiation** models the event when endothelial cells (ECs) leave the parent vessel in order to grow a new sprout. It is a stochastic process that adds new vessel segments to the existing network. Lattice sites occupied by the existing network are visited in random order and at each of these sites a new segment is attached with probability 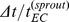 provided that the following conditions are met: the growth factor concentration is non-zero, the distance to the next branching point is larger than 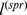 and the time spent within the tumor is less than 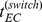. The new segments are created along neighboring lattice edges where the growth factor gradient is maximal and where no other vessels are already present. A vessel is tagged as “within the tumor” if at least one of the endpoints is within the tumor, which is true where the level set function 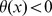 (see below). Vessels also have a property which can tag them as sprouts and tell for how long they have been sprouts. We denote this “life-time as sprout” as 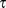.

**Sprout migration** is the process in which initial sprout vessels continue to grow. The probability is 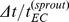 for vessels which are tagged as sprout. A growth event is realized by appending a vessel segment along a single lattice edge in the same direction as the existing sprout. Sprout vessels are untagged and become normal vessels if the tip fuses with another vessel such that blood can flow or if their 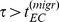, where 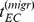 is a parameter which defines the maximal sprout growth time. If the tip fuses with another sprout without creating a conducting branch then it remains tagged as sprout. Sprout initiation can also start from sprouts which emulates tip splitting as observed in-vivo and in-vitro. Sprouts are excluded from the collapse, degeneration and circumferential growth mechanisms.

**Wall degeneration** models the detachment and disintegration of cell layers and membranes around the vessel lumen. Therefore we implement the property 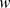 which reflects the vessel wall thickness for normal vessels, and continuously decreases for tumor vessels with the rate 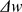 until zero. For values smaller than e.g. the size of an EC, 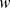 becomes an abstracted representation of the stability and tightness which the remaining EC layer provides. 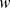 is initialized (sprouts and initial vessels) with the wall thickness of real healthy vessels in dependence on their radius [24] (see Supplement S1).

**Vessel collapse** models pinch off of blood flow and complete disintegration of the vessel. It is a stochastic process where a vessel can be removed with probability 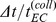 under the condition that its wall stability variable 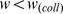 and its wall shear stress 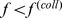. Thereby 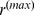, 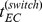, 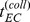, 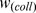 and 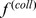 are model parameters. Recently the Ang-Tie system was modeled in a similar context [25]. This is straight forward to include in future work. Here we model the effects of it, rather than the system directly.

**Vessel dilation** models the switch to circumferential growth within the tumor [21]. During circumferential growth the vessel radius increases continuously with the rate 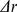. The requirement is hereby that 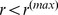, the average growth factor concentration over the segment is non-zero and the time spent within the tumor is larger than 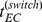.

### Initial Blood Vessel Network Construction

To our knowledge there are no data sets from real networks available that cover a few millimeters of tissue and represent the complete vasculature including micro vessels in a form which is convertible to a “network of pipes” as required for our modeling purposes. Therefore we decided to generate it algorithmically. Our aim is to maximize the lattice occupation with a network which exhibits a hierarchical topology and homogeneously distributed capillaries.

A well known method is constraint constructive optimization (CCO) [26] in which a tree is grown by successively adding branch segments at locations given by some optimality criterion e.g. minimal total surface area. The constraints are that there is no geometrical overlap of the branch segments and that new segments must reach certain previously unperfused tissue blocks. The radii at branching points 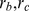 behave according to Murray's law [27] 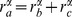, where 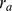 is the radius of the parent branch and 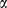 is an exponent. 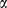 has been found to range between 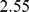 and 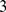. The latter is a common choice which is also taken here.

In [28] a method was presented in which vascular trees on a lattice are stochastically grown and remodeled. This has the advantage of being relatively simple and being capable of building connected networks comprising arteries, capillaries and veins, whereas in CCO one typically has “dangling” terminal branches where capillaries should connect to. In [18] we adopted this method in order to obtain initial networks for 2d simulations. Later we applied it on a cubic lattice [19] and here we apply it on a FCC lattice. An illustration of the steps can be found in Supplement S1.

The initial network construction is based on a relatively coarse lattice with a lattice constant that corresponds to the mean inter-capillary distance 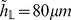. After the construction of the network on the coarser network it has to be mapped on the finer lattice with 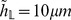 for use in the subsequent simulation. FCC lattices can be subdivided not unlike cubic lattices, meaning that sites and edges of the coarse lattice coincide with site and edges on the fine lattice. The tedious details are omitted here.

The construction is initialized by placing nodes which serve as roots for the trees onto boundary sites of the lattice. The type of these nodes is either arterial or venous, placed in alternating order. The subsequent construction is then carried out in two stages. In stage one, trees are grown by a stochastic process in which “structural elements” are successively appended to one of the current tree leafs. As structural element we take either single vessels or a Y-shaped aggregate of three vessels. The element, its orientation and the leaf are determined by randomly (see Supplement S1). Eventually the lattice is filled but arterial and venous side are not interdigitating sufficiently to yield a homogeneous capillary distribution.

This is corrected in a second remodeling stage. Capillaries are temporarily inserted in-between neighboring arterial and venous terminals. We set the capillary radii to 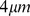. Radii of terminal branches are set to 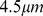 for arteries and 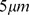 for veins. Radii of higher level vessels are determined by Murray's law. As a result an intermediate functional vascular tree is obtain for which blood flow is computed. Shear-stress dependent growth and shrinkage is carried out by stochastic removal and attachment of vessels from or to terminal branches. High shear-stress means higher probability to grow and vice versa. The idea originates from the observation that high shear stress indeed promotes vessel survival and stability [29]. We repeat this stage until the number of capillaries reaches a plateau. Trees can potentially grow from each of the root nodes. A few of these trees establish themselves while most of them regress and disappear.

For this paper we extended the algorithm from [18], [19] with an “outer” loop producing increasingly fine resolved networks in a hierarchical fashion. This effectively reduces the tortuousity of major vessels. The first level (coarsest network) is constructed as described above. Then the lattice is refined, halving the lattice spacing and doubling the number of sites in each direction. Arteriovenous trees are kept in place and capillaries are discarded. Each vessel segment now occupies two lattice bonds. The lattice spacing is then reset to its former value. Hence, the spatial extend and segment lengths are effectively doubled. Now the random growth and remodeling steps are executed as above, where the previous terminal nodes now serve as new roots. This up-scaling and growth procedure is repeated a preset number of times. The results shown here were generated from a 25
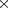
31
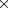
31 base lattice and 2 up-scaling steps.

### The Continuum Model for Tissue

Our tissue model is based on the framework developed in [30] which describes the tissue as a mixture of various tissue constituents. A mathematical model is formulated in terms of smoothed fields of quantities such as density, velocity, stress, etc. Several constituents can coexist at one material point due to smoothing. Assuming incompressibility, one can describe the composition in terms of volume fractions with the constraint that the fractions sum up to one at every point in space. For brevity, we just give the final set of equations. A derivation can be found in [30], see also [31]. The result is a system of partial differential equations of the diffusion convection reaction type.

First, let us denote tissue constituents and their volume fractions:

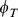
 : tumor cells

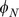
 : normal cells

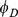
 : necrotic cells

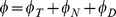

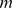
 : ECM

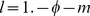
 : interstitial fluid

Tumor cells and normal cells are immiscibly separated by the interface 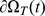, where 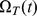 is the tumor region.
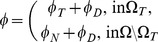



This is analogous to immiscible liquids, where cell-cell adhesion forces correspond to the atomic forces in the liquids. We assume however that the adhesion forces are very weak, which allows us to neglect the surface tension term which would normally appear in the momentum balance equation. It will be included in future work.

All 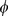 constituents move with the same velocity field 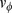 which is driven by the gradient of a solid pressure (the isotropic component of the stress tensor) 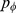. It is based on the assumption that inertia is dominated by friction against a rigid ECM through which cells flow like a liquid through a porous medium. Therefore we have
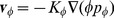
(1)

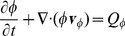
(2)

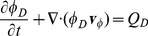
(3)

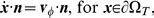
(4)where (1) is the condensed momentum balance, 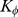 is a mobility constant and 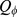 and 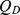 are source terms to be defined below. Note that this set of equations is applicable to tumors that have a clearly delineated rim as for instance rat C6 gliomas and human glioblastomas [21], human malignant melanoma [20], leiomyomata [32], etc. It is not valid for non-solid cancers like Leukemia and highly invasive tumors which do not have such a clearly delineated rim.

The motion of 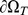 is formally defined by (4). In practice we use the level set method [33] to represent 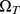 and 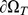 and introduce an auxiliary field 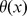 which gives the closest distance to 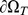. It is signed so that 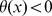 for 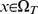. Over time it evolves according to the advection equation
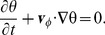
(5)


We can now define 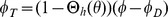 and 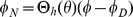, where 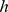 is the lattice spacing of the numerical grid (see below) 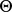 denotes a smoothed Heaviside step function with width (see Supplement S1).

For the pressure we take
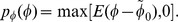



For simplicity and the lack of better knowledge we use a linear elastic law with elastic modulus 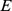. Also, we assume that cells do not exert pressure upon each other when 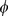 is less than the volume fraction in a fully relaxed state 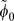.

The source terms are composed of contributions from 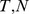 and 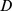 as follows
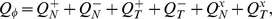


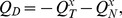
where 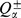 stands for proliferation and apoptosis of phase 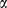 and 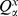 stands for necrosis.

We assume proliferation depends on packing density [34], i.e. volume fraction 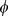, and on available nutrients 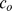. Cells do not proliferate in regions with high density where apoptosis reduces the density towards the so called homeostatic (equilibrium) density 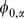. At 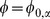, and for sufficiently high 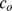, apoptosis and proliferation rates cancel so that the net production rate 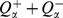 vanishes. Moreover 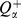 varies linearly with 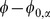. Under low nutrient conditions proliferative activity stops, i.e. 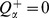 for 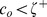, where 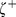 is a threshold parameter. Consequently, apoptosis and possibly necrosis reduce the cell density. The difference between these events is that apoptosis leaves no debris as cells are deconstructed in an orderly fashion, i.e. the respective cellular material vanishes. Necrosis occurs under very low nutrient conditions if 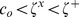, where 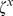 is also a threshold parameter. The fraction of cells undergoing necrosis is transferred to 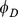 via the rates 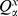. In total this behavior is summarized in the following formulas:
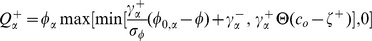


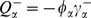


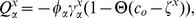
for 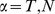, where 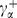, 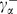 and 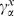 are constant rate coefficients (proliferation, apoptosis and necrosis), 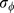 determines the sensitivity to density variations and 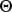 is the Heaviside function. Note, the use of “
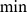
” in conjunction with the Heaviside function. It limits the proliferation rate by either 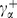 or 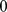 (no proliferation) depending on nutrients.

### Interstitial Fluid Flow

IF is commonly modeled as a liquid within a porous medium, e.g. [11], [12], [14], [35]. We follow this approach and assume that cells and ECM collectively constitute the porous medium. Here we consider only stationary states, with a static tumor and a rigid medium, thus 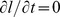. Mass balance for the IF fraction 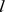 requires that
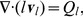
(6)with its velocity 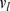 and source distribution 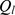. Neglecting inertia terms one obtains the momentum balance equation
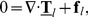
(7)where 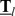 is the stress tensor of the liquid, 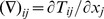 and 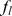 an interaction force with the other constituents. We use the results in [31] and [30] where constitutive relations for 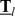 and 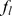 were obtained for the case of a solid-fluid mixture. Assuming that the interstitial fluid is an ideal inviscid liquid, its stress tensor consists only of the contribution from the interstitial fluid pressure pressure 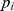 or in the following just 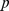.



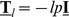
(8)The interaction force is defined in such a way that we later obtain a variant of Darcy's law
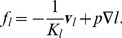
(9)


The first term represents friction with cells and ECM fibers, where 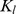 is a tissue dependent permeability coefficient. Substitution of equations (8) and (9) in (7) yields a variant of Darcy's law
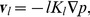
(10)which leads to an elliptic equation for the pressure



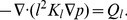
(11)Note that 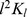 is the classical conductivity of the porous medium. We define 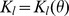 so that it smoothly interpolates between parameter values for tumor 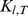 and normal tissue 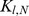. 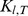 and 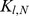 are chosen so that the conductivity in the bulk assumes experimentally determined values. Note that 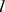 is almost constant distal to the tumor boundary and varies over a small value range since 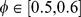.

The source term is composed of contributions from the vessel network 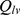 and lymphatic sinks 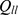 so that 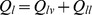. Both are determined by the flux across the channel walls. For vessels, this flux is driven by the pressure difference 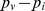 and an osmotic contribution 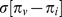 (Starlings equation) [36]. For lymphatics we assume an analogous relation but neglect osmosis due to the lack of data.
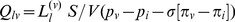
(12)

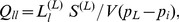
(13)where 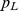 is the lymphatic pressure, 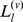 and 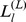 are permeabilities, 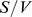 and 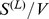 are the channel surface area densities per volume and 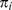 and 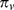 denote the so called oncotic pressures. 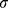, the reflection coefficient, is a tissue specific value.

The standard approach for modeling exchange with vessels on a small scale would use boundary conditions at the vessel walls, while tessellating the surrounding space with a fine grained mesh. However this would make the large length scale which we are interested in inaccessible due to the size (we have of the order of 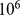 vessel segments). Instead we integrate the flux approximately over the vessel surfaces within each numerical grid cells and add it as source term. An approximation inherent to this method is that the space covered by the vasculature is not excluded from the interstitial space.

Hence (12) is not applied in this exact form. The source flux is implemented as superposition of smoothed delta functions 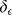 (see Supplement S1 ). Their locations 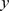 are generated from a stochastic uniform sampling of the surfaces of the cylindrical pipes which make up the vessel network. We write this formally as 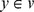, where 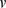 symbolizes a vessel. For a numerical grid cell with index 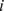 and center 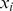, 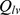 then becomes
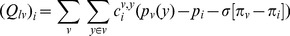
(14)

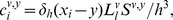
where 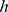 is the grid spacing, 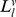 the wall permeability, 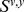 is the area corresponding to a sample on 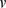, and 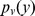 is the blood pressure in 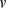 at position 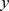, linearly interpolated between the nodes.

Different degrees of vessel leakiness are incorporated based on the maturity state 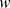. We assume that 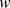 reflects the vessel wall thickness for sufficiently large vessels and that the wall's resistance 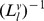 increases proportionally to the wall's thickness. This eventually leads us to
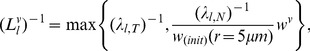
(15)where 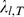 and 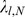 are experimentally determined permeabilities for capillaries in tumor and normal tissue, respectively, and 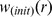 is a formula based on experimental data [24] from which we obtain the physiologically normal thickness of the vessel wall depending on the radius (see Supplement S1 (2)). For small 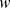 the identification with the wall thickness breaks down and it becomes a mere abstract quantity inversely related to the amount of leakiness. In order to obtain realistic permeabilities for tumor vessels as well, we are therefore free to bound 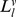 from above by 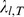.

Lymphatics on the other hand are modeled as continuous sink distribution, where their surface area 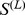 depends on the tissue type via 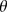 analogous to 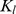 and moreover 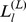 and 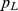 are assumed to be constant. Hence we can use (13) directly in the numerical implementation.

### Chemical Concentration Fields

The basis for the description of dissolved chemicals is the following diffusion convection reaction equation which determines the evolution of the concentration 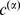 in constituent 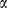 [30].
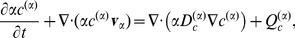
(16)where 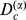 are effective diffusion constants (assumed to be scalar) and 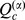 a source term. For nutrients and growth factors, we approximate the concentration as the equal in all phases 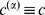 under the assumption that the exchange among constituents is very fast. Then, summation of (16) over all 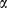 gives
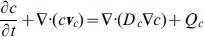
(17)

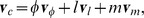
where 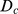 is the composite effective diffusion coefficient, and 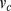 the composite velocity of the mixture. In the following we will use subscripts to 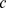 to denote specific chemical species: 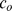 denote nutrients, 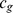 are growth factors. For drug we distinguish concentrations in two different compartments 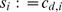 for which 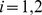 denote the extra-and intra cellular space, respectively.

*Nutrients* are represented by the most prominent one, namely oxygen with its concentration 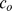. The time scale on which 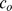 relaxes after changes is negligible, of the order of seconds, and thus 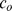 is assumed to be always in a quasi stationary state, instantaneously adapting to changes in the system. Convection is neglected due to the dominance of diffusion. Consequently we obtain
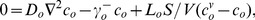
(18)where we already replaced 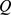 with a particular form of the source term: The second term represents consumption with the tissue dependent rate 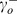. The third term represents the diffusive flux across the vessel wall, which we treat analogous to the interstitial fluid source term (14), only 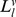 is replaced by 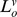, and 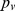 by the blood oxygen concentration 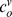. Since we already assumed that the hematocrit is constant over the whole vasculature, we further assume for simplicity that the oxygen concentration 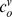 also constant over the perfused parts and zero in unperfused vessels.

*Growth factors* are collectively represented as a single diffusible species with its concentration 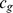. A prominent representative is VEGF which is over expressed in under-oxygenated tumor cells. We assume a constant production rate by tumor cells in locations where 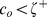 and that it diffuses, binds and degrades everywhere equally. Instead of solving a diffusion equation we use a simpler and faster approximation based on a Green's function approach: every source site generates a linearly decaying contribution to 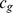 with the cutoff or diffusion radius 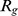. Thus we have
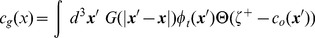
(19)where we define 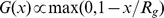, with a normalization constant so that 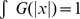. Note that consequently, by definition of the angiogenesis rules, sprouting occurs within 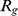 of oxygen deprived TCs and a 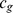 gradient arises along which sprouts are oriented.

*Transport and uptake of drug* is modeled as diffusion advection process in the interstitial fluid and sequestration into the cell constituent. We distinguish between the concentrations 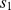 in the IF and 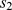 within cells as average over the solvent volume. The tissue volume average reads 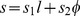 with the volume fractions 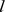 and 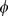 as defined above. Following (16), we define specialized mass balance equations as
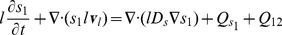
(20)

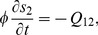
(21)where 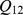 is the exchange rate between the two compartments, 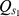 the source contributions from vessels and lymphatics and 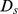 the diffusion coefficient in the IF. For a simple derivation of 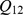, we assume the total flux of molecules across the cell-fluid interface area 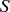 within some volume 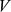 has the form 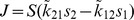 with the rate constants 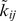 which model the combined effect of diffusion through the cell membrane and intracellular binding and unbinding. We write 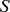 in terms of the single cell volume 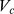 and surface area 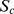 as 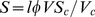, assuming that only the fraction 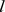 of the cell surface is in contact with the IF. Then we obtain with 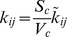


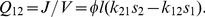
(22)Furthermore the contributions from vascular and lymphatic exchange are given by
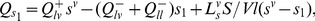
(23)where 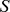 has the original meaning of vessel surface area again. The diffusive permeability 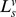 is defined exactly like 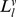 in (14) and (15) with correspondingly exchanged subscripts including the permeabilities of tumor vessels 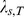 and normal capillaries 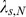. 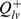 stands for extravasated fluid volume per mixture volume carrying the concentration 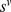 which is the concentration within the vessels. We assume that 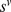 is homogeneous over the whole network but time dependent where the dependency is given as closed formula e.g. an exponential decay after a hypothetical injection at 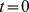. 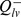 stands for fluid uptake by vessels. Analogously 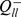 for uptake through lymphatic. Since these terms represent flow out of the interstitial space, they are multiplied by 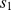 in order to obtain the respective solute flux. We could define a 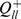 for symmetry but in practice fluid always flows into lymphatics, never in the opposite direction. We treat 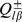 analogously to 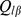, for 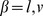 in (12) and (13) with the exception that only contributions are added where the blood or lymphatic pressure is larger (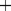) or lower (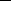) than the IF pressure. Indeed 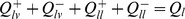.

### Numerical Implementation

Solutions to partial differential equations are computed by finite difference schemes on a regular uniform staggered grid [37]. Numerical values for concentrations, volume fractions, etc. are defined on grid cells, while velocities and fluxes are defined on faces. The grid spacing 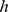 is 30 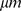 which corresponds approximately to two to three tissue cells. The diffusion terms are discretized by standard 9 point centered difference stencils. All system of linear equations are solved with (algebraic multigrid - if needed) preconditioned conjugate gradient method. Specifically, we use the solvers in [38]. Advection terms are treated by a central scheme for conservation laws [39]. In the time, the operator splitting technique [37] allows treatment of various sub-systems separately, i.e. sub-systems are advanced one by one, always using the newest available state. The cell volume fractions 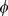 and 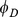 are updated simultaneously with the 2nd order improved Euler method. The level set function 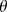 is updated likewise. The vessel network is updated in 1 hour steps. In these periods for 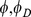 and 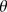 smaller sub-steps must be taken the length of which is dictated by the stability conditions of the time integration methods. In practice these steps are about 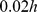 wide. Sometimes 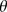 must be “redistanced” in order to restore the distance function property 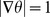. The WENO method presented in [40] works very well for our purposes. The computation of 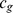 and 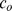 as well as redistancing are not performed every step. We determine the time between updating these fields by the time it takes tissue cells to cross a numerical grid cell and also by the time scale of the source term, which gives the time 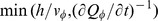, where we take the minimum over space and time since the last update. The numerical solution of the drug concentrations 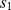 and 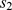 is carried out using the central advection scheme [39] and the improved Euler method.

### Parameters

A list of parameters for our base case system can be found in tables 1 and 2. The sprouting parameters 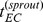 and 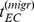 are estimated from in-vitro endothelial cell (EC) migration experiments in [41]. It is known that angiogenesis is inhibited in ECs near existing branching points. For this 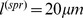 seem reasonable, which are about two nearby ECs. The vessel dilation rate 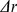 and maximal radius 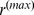 was extracted from [21] where the spatial compartmentalization of human melanoma was described. The wall thickness 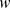 is initialized at 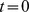 depending on the vessel radius (see Supplement S1 (2)) guided by experimental data [24]. The wall degradation rate was estimated from [21] based on tissue section at increasing stages of tumor progression. We simply observed how long it takes until the supporting wall structures of a vessel with a certain radius are removed. For the critical collapse shear stress 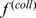 we assumed that it is a low percentage of the average shear stress within the system, also guided by comparison of predicted vascular density levels with data from [20].

**Table 1:** Model Parameters: Tumor Growth.

Parameter	Value	Unit	Description
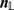	(825,990,961)		Lattice size
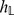	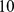	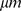	Lattice spacing
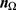	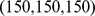		Lattice size
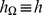	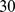	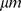	Lattice spacing
	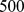	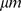	Initial tumor diameter
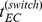	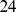	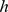	Circumferential growth switch delay
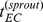	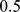	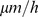	Sprout extension speed
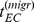	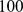	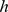	Sprout activity duration
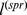	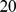	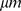	Sprout sites minimum separation
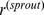	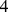	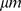	Initial sprout vessel radius
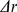	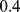	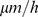	Vessel dilation rate
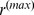	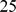	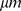	Maximum dilation radius
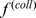	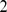	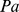	Critical wall shear-stress
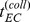	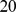	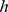	Unstable vessel survival time
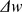	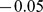	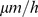	Vessel wall degradation (w ) rate
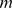	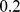		ECM fraction
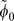	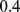		Relaxed cell fraction
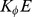	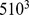	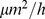	Cell mobility 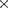 elastic modulus
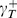	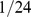	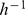	Tumor cell proliferation rate
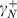	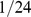	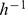	Normal cell proliferation rate
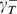	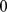	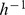	Tumor cell apoptosis rate
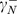	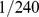	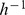	Normal cell apoptosis rate
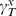	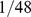	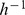	Tumor cell necrosis rate
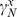	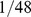	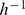	Normal cell necrosis rate
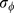	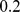		Cell pressure sensitivity
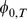	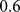		Homeostatic tumor cell fraction
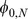	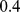		Homeostatic normal cell fraction
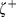	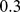		Oxygen threshold for proliferation
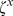	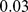		Oxygen threshold for necrosis
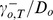	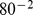	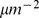	Oxygen consumption rate in tumor
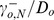	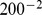	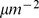	Oxygen consumption rate in normal tissue
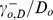	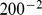	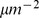	Oxygen consumption rate in necrotic tissue
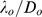	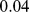	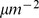	Vessel permeability to oxygen
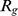	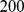	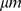	Growth factor diffusion range

List of parameters for tumor growth.

**Table 2:** Model Parameters: Interstitial Fluid and Drug.

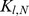	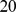	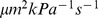	Normal tissue permeability coeff.
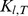	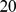	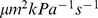	Tumor tissue permeability coeff.
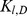	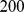	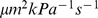	Necrotic tissue permeability coeff.
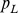	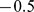	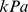	Lymphatic fluid pressure
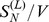	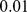	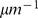	Lymphatic surface area per volume in normal tissue
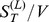	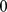		Lymphatic surface area per volume in tumor tissue
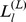	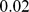	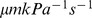	Lymphatic wall permeability
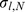	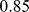		Osmotic reflection coefficient in normal tissue
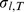	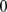		Osmotic reflection coefficient in tumor tissue
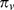	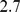	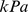	Vessel oncotic pressure
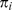	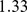	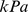	Interstitial oncotic pressure
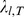	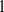	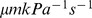	Tumor vessel wall permeability
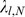	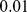	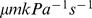	Normal capillary wall permeability
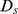	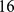	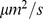	Drug diffusion coefficient
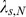	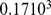	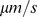	Vessel permeability to drug in normal tissue
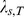	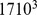	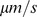	Vessel permeability to drug in tumor tissue
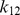	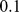	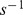	Drug transport rate, extra-to intracellular
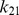	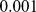	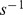	Drug transport rate, intra-to extracellular

List of parameters for interstitial fluid flow and drug delivery.

The oxygen level in our model is dimensionless and normalized to 1 which is the level within vessels. We divide the diffusion equation (18) by 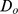. Hence it is left to determine the quotients with the consumption rates 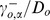 and vessel permeability 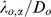 for 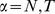. For this purpose we use that the penetration depth (i.e. the length scale on which the solution decays around vessels) in tumor tissue is about 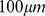 and can be expressed as 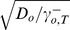. We assume that 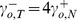. The precise number is arbitrary and non-crucial but reflects that tumor cells have a higher oxygen consumption rate leading to decreased tissue oxygen levels. We then tuned 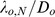 so that the oxygen level in normal tissue is above ca. 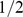. For simplicity we assume that the permeabilities in tumor and normal tissue are equal 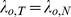.

We assume that tumor and tissue cells have the same fastest possible proliferation rate (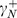 and 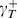) of once per day. The time to live for normal cells is assumed to be 10 days after which they undergo apoptosis, yielding 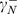. Tumor cells have acquired mutations which enable them to circumvent the apoptotic mechanisms. Therefore we set 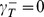. Cells under severe hypoxia are assumed to die off relatively quickly within 48 h (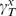 and 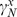) and become necrotic tissue.

The oxygen threshold below which cells become necrotic is 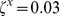. Cells stop proliferating when the oxygen level is below 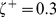 which is ca. 60% of the lowest level in normal tissue. Since only tumor cells are ever exposed to low oxygen levels, we also do not distinguish between tumor and normal tissue here.

Our cell volume fractions reflect a high-density prototypical tissue. We assume that tumor cells have become less sensitive to solid pressure from nearby cells and so their homeostatic level is 0.6 (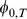) while it is 0.4 (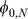) for normal cells. Here we follow [34], where the idea for this pressure regulated proliferation originated. A similar model was also employed in [42] but with different parameters.

Parameters for interstitial fluid flow and drug transport are summarized in table 2. The permeability coefficients 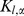 and 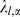 as well as osmosis parameters 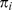, 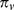 and 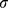 are obtained from [35] and the references therein. Where the actual permeability is provided, e.g. 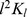, we compute the coefficient by dividing with the typical 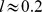 in the respective tissue. For lymphatics we assume a wall permeability (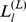) which is of the same order of magnitude as for capillaries (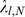). The lymphatic surface area per volume (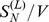) is estimated by assuming that there is a grid-like network with a channel every 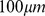 and a radius of 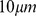. We leave the tumor without lymphatics (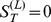), since these are absent or dysfunctional in tumors (see [5] and the references therein).

The drug distribution model is guided by experimental data on the pharmacokinetics of Doxorubicin, which has been used for a long time to treat various cancers. For the diffusion coefficient 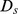 and exchange rates 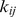 we follow [13] who presented a similar model with additional cellular compartments. The diffusion constant stems from [43] where it was estimated as ca. 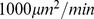, which we use as well. Given an isolated system with two compartments and transition rates 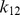 and 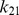, the steady state concentration ratio equals the ratio of the rates. In experiments with cell cultures [44], intra-cellular to medium ratios of ca. 100 were observed. It seems reasonable to associate 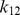, the cell uptake rate with diffusion through the vessel wall. In [13] this rate was estimated as 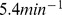. Thus we simply use 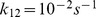 and 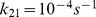. Note that 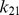 is close to the estimated lysosomal release rate which is also the slowest rate in the model proposed in [13], so this may be identified as bottleneck for release. It remains to determine the vascular permeabilities. We estimate these based on the diffusivity in plasma. As an approximation we write the permeability of a planar sheet of thickness 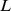 as 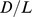, where 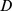 is the diffusion constant. We take 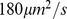 for 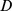 in plasma and we identify 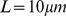 with the thickness of the capillary wall. We assume that drug only diffuses through the gaps between endothelial cells (ECs). Assuming that in very leaky tumor vessels, there would be a circular gap with 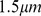 radius per EC [35], we arrive at the fraction of gaps over the vessel surface 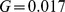, assuming 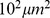 per EC, thus 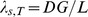. Assuming the difference between tumor and normal capillaries is due to leakiness, we determine 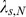 by requiring that the ratios are equal: 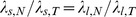.

## Results

### Tumor Growth

**Figure 2:**
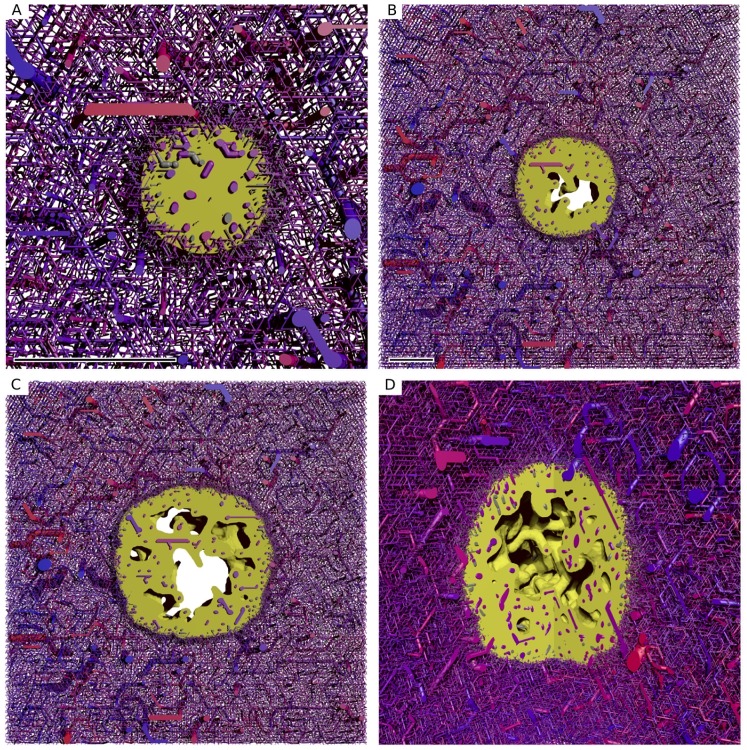
Snapshots from the simulation of a growing tumor. (A) to (C) depict 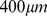 thick slices through the origin. The scale bar indicates 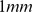. (A) is a close-up. (B) and (C) have the same scale. The snapshots are taken after 100 h, 400 h, 700 h. (D) shows the same time as (C) from a different point of view where a quadrant was cut out. The boundary to the viable tumor mass is rendered as solid yellow surface. Necrotic regions appear as void spaces within the tumor. The blood vessel network is rendered as collection of cylinders, color coded by blood pressure. Red is high (arteries), and blue is low (veins).

Snapshots from a simulation are displayed in Figure 2. We performed 15 simulation runs, producing 15 final states which differ in their initial blood vessel networks (and the seeds for the random number generator used for the stochastic events during the simulation). For a video visualizing the spatiotemporal evolution of the model see Supplements S14 and S14.

**Figure 3:**
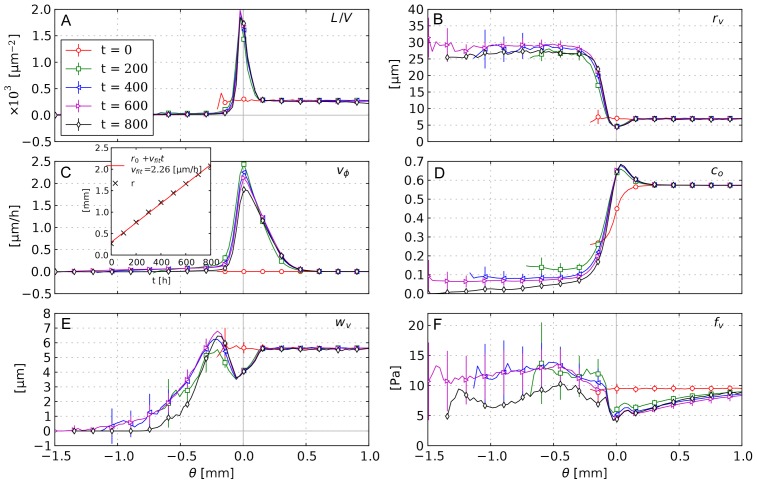
Radial distributions of biophysical properties in the tumor growth model. (A) MVD, (B) vessel radius, (C) cell velocity, (D) oxygen, (E) wall thickness, (F) wall shear stress. The distributions are plotted vs. the distance from the tumor surface 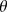. Each data point corresponds to the average over a small 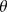-interval. The errorbars indicate the standard deviation among different simulation runs (see text). The inset in (C) shows the approximate radius of the tumor and a linear fit. This radius is determined by averaging the distance from the origin over numerical grid cells where 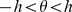.

Initially, the tumor is prepared as a small sphere in which the tissue consists of tumor cells instead of normal cells. We define the distance function 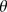 at 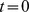 as the signed distance from the sphere boundary. The tumor is located in the center of the simulation box and has a radius of 0.5 mm. Increased oxygen consumption leads to decreased oxygen levels within the tumor which leads to expression of growth factors which again stimulates angiogenesis within 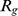. Eventually blood-perfused neovasculature raises the oxygen level in the tumor periphery and enables further tumor growth. The first snapshot in Figure 2 shows the system after 100 h. At this point the system is in a state displaying the typical compartmentalization into high micro vascular density (MVD) rim, decreasing MVD toward the tumor center, isolated vessels threading the tumor, necrotic regions associated with unvascularized regions and tumor proliferation confined to its rim. The tumor continues to grow by vascularizing and pushing into the surrounding tissue, leaving a torturous chaotic tumor network behind. The final snapshot is taken at t  = 700 h where the tumor has reached the edge of the continuum domain 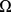. Its final radius is ca. 2.5 mm. By design of the tumor-vessel interactions similar observations were reported in earlier work in [15], [16], [18], where much simpler tumor models were used. See also Figure 3 where important system variables as a function of the distance from the invasive edge 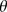 are shown.

To generate these plots we sorted data points based on their spatial position into bins, or shells surrounding the invasive edge according to their 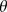 value. The width of the bins is 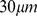. Unless stated otherwise, we computed the averages of the binned values for each simulation run. The plotted data displays the means and standard deviations of the ensemble, not the spatial fluctuations. Spatial fluctuations can be seen in the map plots and are analyzed in more detail only for the distribution of drug. Note that we may define averages over (parts of the) vessel network formally as line integrals over the vessel center lines divided by the total length of respective parts. In practice we generate sampling points on center-lines and sort these into bins as we do with numerical grid values. This is applied e.g. in Figure 3B (vessel radius).

The predictions described above are in good agreement with experimental data from [20] and [21] for human melanoma xenografts and gliomas respectively. Our new continuum model describes tissue more realistically by the incorporation of actual host tissue cells, cell motility and cell-cell adhesion. We will report a more detailed analysis of the resulting morphological aspects elsewhere.

### Interstitial Fluid Flow

Pressure, velocity and source terms were computed numerically for the final tumor configurations at t  = 700 h. For the IF flow studies we assumed that the tumors are static from this time on. Generally the motion of the IF is coupled to the motion of the other tissue constituents since “empty” spaces are filled with the IF. However in our case the velocity of the fluid is orders of magnitude larger than the velocity of cells, for which reason we can neglect these interactions.

**Figure 4:**
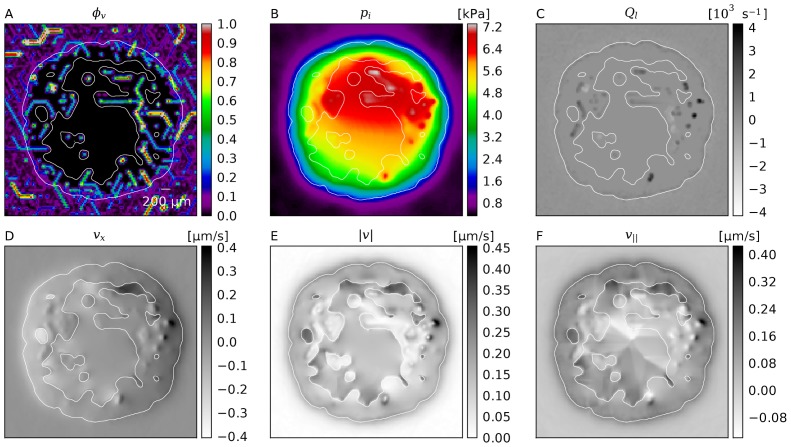
Snapshots of interstitial fluid flow quantities. (A) Vessel volume fraction, (B) IFP, (C) Fluid source term, (D) x-component of the IF velocity, (E) Magnitude of the IF velocity, (F) Projection of IF velocity onto the outward direction, i.e. onto the gradient of 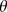. The plots were generated from 2d slices through the center of a typical simulation result from the base case. The contour line indicates the boundary of the viable tumor mass. The internal regions consist of necrotic tissue, while the outer area is normal host tissue.

Figure 4 shows slices through simulation data of one sample. Figure 4A displays the vessel volume fraction 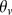. The data are generated by superposition of smoothed delta functions which are distributed stochastically within the cylindrical volumes comprising the network edges. In Figure 4C we plotted the source term 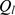 which is the IF volume flowing in or out of the interstitial space per volume and time. By definition, lymphatics are absent within the tumor, thus therein the only sources and sinks are blood vessels, which appear as lengthy blobs with positive (extravasation) or negative (uptake) contributions. Uptake is possible since the blood pressure can also be lower than the local IFP. At the tumor rim we see a significant amount of fluid being taken up, since there is a strong outward flux from the tumor which is absorbed into lymphatics and potentially also into parts of the neovascular plexus.

**Figure 5:**
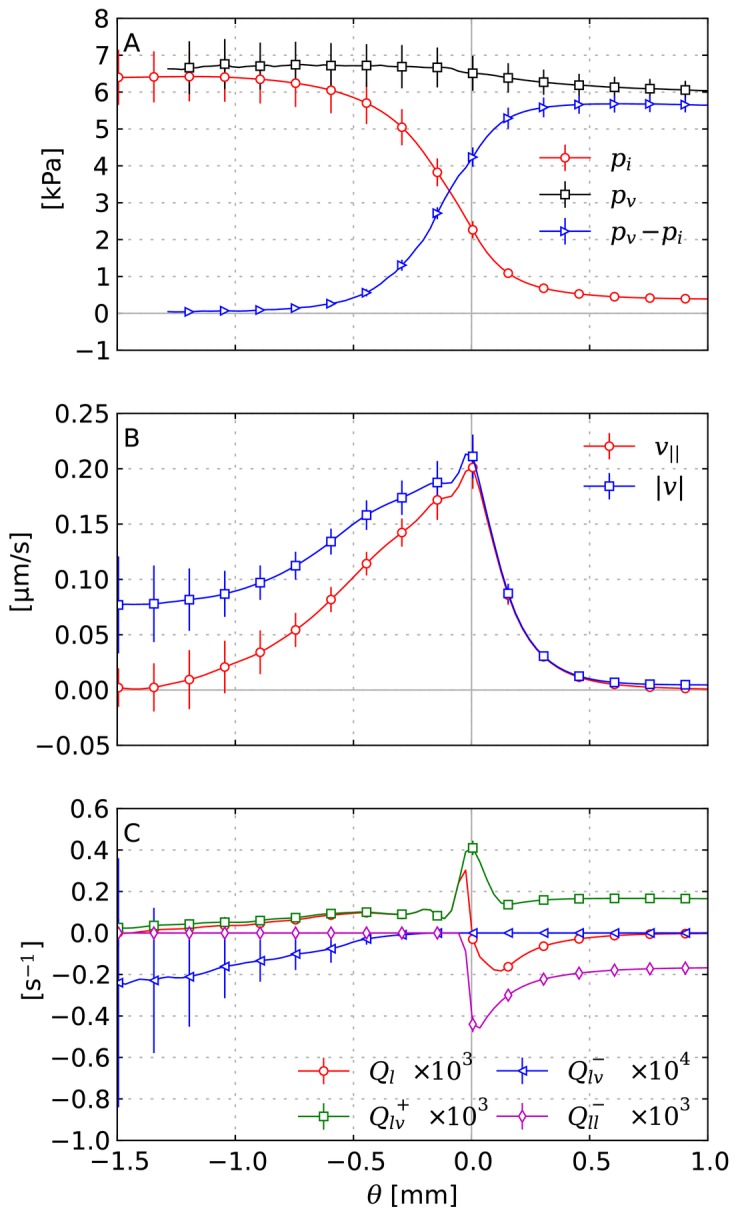
Radial distributions of interstitial fluid flow quantities. (A) IFP, blood pressure and the pressure drop across the vessel wall. (B) IF velocity: 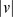 plots the magnitude and 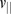 the projection onto the direction of the shortest path to the tumor boundary 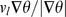, respectively. (C) The total fluid source term 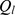 as well as contributions to it which are: extravasation 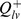, uptake by vessels 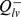 and uptake by lymphatics 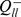. Each data point corresponds to the average over a small 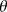-interval. The error bars indicate the standard deviation among different simulation runs (see text).

The IFP profile is elevated within the tumor and decays rapidly over its boundary. See Figure 4D and Figure 5A. The peak pressure in the tumor center is ca 6 kPa (45 mmHg). Outside it is 0.5 kPa (3.75 mmHg). We set the lymphatic pressure to −0.5 kPa, and the average blood pressure is ca 6.25 kPa (47 mmHg). In models using pure capillary networks a pressure range from 15 to 25 mmHg is commonly either directly set or imposed via boundary conditions, e.g. in [10], [12], [14], [15]. In the presence of higher level arteries (as in our model) a much higher IFP can be observed, because of the elevated pressure in arteries and connected vessels. Mean in vivo tumor IFPs are reported in [35] from 0 to 5 kP (38 mmHg) for various tumor types. Most tumors exhibit a high degree of heterogeneity, with deviations from the mean of 100%. The elevated IFP has a finite “penetration depth” into normal tissue (see below). Beyond that the IFP appears relatively homogeneous. Fine grained fluctuations are introduced by fluctuations of the capillary surface area per grid cell.

The order of magnitude of the interstitial fluid velocity is considered to be 0.1 to 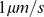 [4], [45]–[47]. In particular, it is found to be highest close to the tumor boundary where the pressure gradient is steepest, leading to a strong outward flux. Our results, shown in Figure 4A and Figure 5B are in agreement with this experimental observation. The velocity peak in the region is at 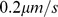.

Furthermore the velocity patterns in our data show a significant amount of fluid being transported in between tumor vessels. In Figure 4D this can be directly observed. For the whole ensemble we measured the outward projection of the velocity vector 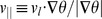 together with the velocity magnitude 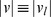, which are plotted in Figure 5. Spatial distributions from one simulation can be seen in Figure 4E and F. Within the tumor center, 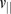 vanishes whereas 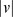 decreases to ca. 0.05 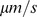. This means that the flow becomes increasingly isotropic toward the center, where the IF apparently flows in between isolated tumor vessels instead of to the tumor boundary. This flow is more than an order of magnitude faster than IFF in normal tissue.

In the tumor periphery fluid uptake by lymphatics is significantly increased compared to normal tissue further away (Figure 5C, 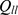) because since lymphatic vessel are absent in the tumor, the extravasated fluid must cross the tumor boundary to be absorbed in normal tissue. The determining equation for the IF pressure 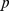, and the equations for oxygen and growth factors, have a structure like 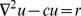, for some constant 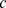, dependent variable 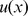 and arbitrary distribution 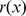. For this equation any local change in 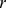 causes an exponentially decaying disturbance in 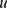. The length scale of the decay is 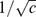. Since 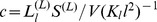 for the IFP, we see a “penetration depth” of 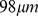.

Furthermore, in addition to the fluid that originates from the tumor interior, we find that the neovascular plexus extravasates a huge amount of fluid (Figure 5C, 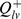). The collective surface area of these vessels is large due to the amount of vessels, they are very permeable and the 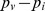 difference is relatively large, so this is not surprising but it has implications for the escape of tumor cells from the rim into the lymphatic system, and subsequently metastasis.

At this point we should discuss the validity of our approximation that neglects the loss of blood plasma from the vasculature due to extravasation. Apart from the study of IF flow this approximation is standard but in particular for tumor blood flow the coupling through leaky thread-like vessels it is a more severe simplification, where one would ideally solve for the IFP and blood pressure simultaneously and fully coupled. To clarify this we first determined the fraction of extravasated fluid relative to the total vascular blood flow into the tumor. To be precise we computed
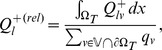
where 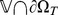 symbolizes the set of all vessels which intersect 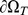 with blood flow directed into the tumor. We obtain 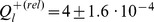 over our base case states, which suggest that a fully coupled solution would not differ significantly from our solution. For completeness, the absolute values are presented in Table 3.

**Table 3:** Tumor volume, blood flow and interstitial fluid sources.

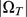	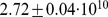	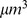	Tumor volume
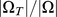	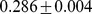		Fractional tumor volume
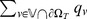	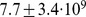	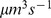	Blood flow through tumor boundary
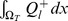	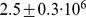	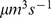	IF influx (tumor)
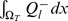	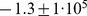	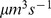	IF uptake (tumor)
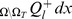	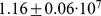	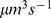	IF influx (normal)
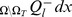	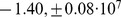	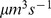	IF uptake (normal)
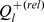	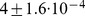		Ratio of IF extravasation to blood flow.

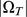
 and the quantities involving 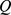 were computed by numerical integration, i.e. summation over grid cells, weighted by 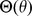 or 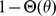 as required by the respective region. Tumor blood flow was computed by summing 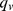 over vessels where (i) the sign of 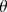 changes between the endpoints, (ii) blood flow is directed into the tumor, which is straight forward to check based on the nodal blood pressures and 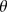. Of course, mass is preserved, i.e. inflow and outflow are equal (in particular since we neglect extravasated fluid). Also due to mass conservation, the IF uptake in normal tissue is slightly higher than influx because flux from the tumor is absorbed as well. Uptake within the tumor is low due to the lack of lymphatics. 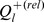 is the ratio of 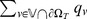 to 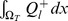, indicating that only a very small fraction of the blood plasma which is entering the tumor is lost into the tumor interstitium.

Moreover, we estimate the length scale over which IFP coupling would cause a decrease of the blood pressure along a single isolated vessel. For this purpose let 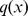 be the flow rate as volume per time through a blood vessel with the axial coordinate 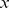. It is determined by 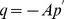, i.e. Poiseuille's law, where 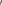 denotes the derivative with respect to 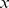, 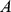 a conductivity constant and 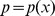 the blood pressure. The fluid loss through the gaps of the vascular walls is the derivative of 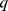, i.e. 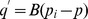, whereby we have incorporated the osmosis contribution 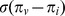 into an effective blood pressure 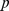. 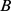 is defined as 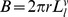, where 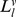 is the wall permeability constant. 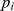 denotes the interstitial pressure.

**Figure 6:**
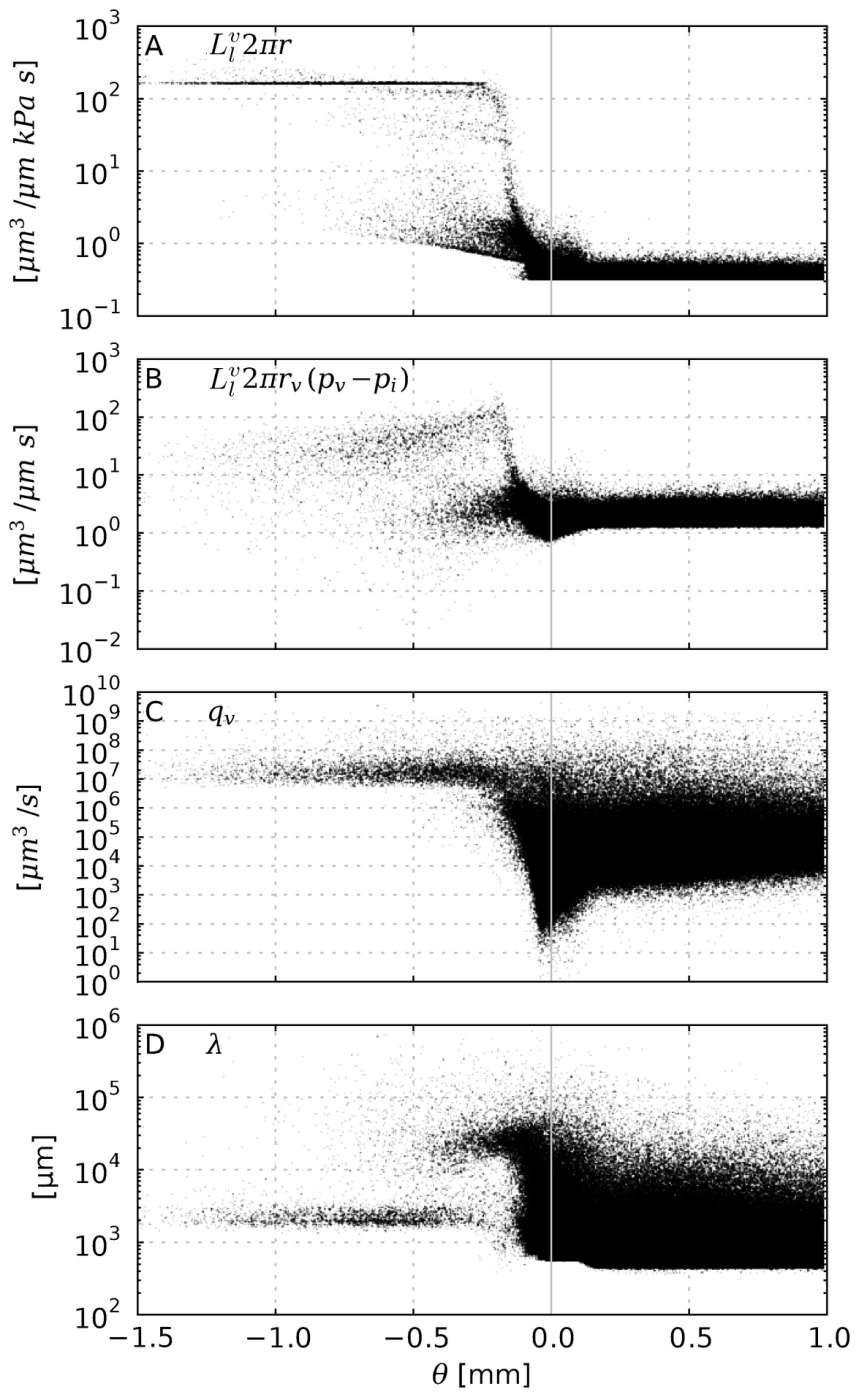
Radial scatter plots of properties related to (trans)vascular flow. (A) the vessel wall permeability multiplied by the circumference 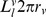 (B) the transvascular flow 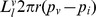, which is the fluid volume that flows through the vessel wall per vessel length and time. (C) the flow rate of vessels 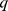, i.e. the blood volume per time that flows through the cross section. (D) an approximate length scale over which 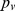 decays toward 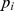 (see text). Data points stem from uniformly distributed sampling points on the vascular networks of 15 simulation runs.

If we now assume that 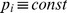, we can easily derive a characteristic length scale 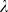 over which 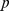 approaches 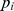. By combining the above equations, we solve for 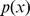 and obtain 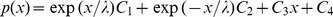, where 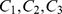 and 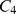 are constants which must be determined by boundary conditions. For 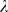 one obtains 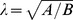 i.e. the root of the ratio of flow to wall conductivity. Note that for 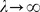 one can recover the standard Poiseuille's law. Based on our model parameters, the order of magnitude of 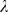 is estimated to lie within 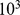 to 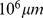, depending on the vessel. Actual measured values are shown in Figure 6D. Within the tumor 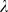 is actually longer than the system size. For capillaries 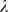 may be around 1 mm, which is still much longer than the length of typical capillaries. The value of 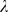 for major normal vessels is similar to tumor vessels. Other relevant variables namely 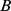, 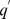 and 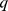 are shown in Figure 6A, B, and C, respectively. In spite of the simplifications used we think this justifies the uncoupled evaluation of the interstitial fluid flow.

In the following we further comment on blood flow and extravasation. In silico data for 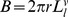 in Figure 6A shows a 20 fold increase from normal tissue to tumor tissue. Remarkably we can distinguish between an increase of 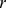 and 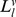. The clustering with short ramp-up is associated with initially thin vessels which dilate to the maximal radius 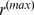. Their permeability 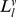 increases simultaneously and eventually hits is upper bound 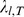. A plateau of maximal 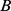 forms where 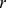 and 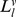 reached their bounds which is here about 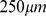 behind the tumor boundary. The lower ramp corresponds to thicker vessels, too thick to dilate (because 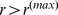), but for which 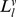 increases.

The flow rate 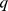, shown in Figure 6C, displays a clear distinction between tumor and normal tissue as do most other variables. In the tumor center it is more uniform and orders of magnitude higher than in normal tissue. 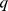 varies with the radius like 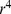, thus dilated vessels provide very well conducting pathways acting as arteriovenous shunts. Most of the data points in normal tissue stem from capillaries which have respectively slow flow rates. Going up the vascular hierarchy, we find increasing flow rates beyond those of tumor vessels and of course less vessels. In the neovascular plexus close to the tumor boundary we observe lower-than-normal flow rates, which is plausible since the blood volume is distributed over more vessels, which implies slower flow velocities in order to to satisfy mass conservation. See also our results and discussion in [18].

The flow rate through the vessel wall 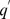, shown in Figure 6B, correlates well with the wall permeability 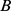 weighted by the vessel circumference. Its magnitude within the tumor is about an order of magnitude larger than in normal tissue. This contradicts the common hypothesis that increased interstitial pressure hinders fluid extravasation. However, the permeability increase dominates the decreased IF pressure difference, which is only halved within the tumor compared to normal tissue.

### Drug Transport

Here we analyze the spatiotemporal evolution of the concentration distribution 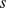 of some substance over the time frame of 96 hours. 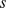 was computed numerically according to (20) based on the data from previous simulations of tumor growth and interstitial fluid flow. Thereby the tumor is considered static and the IF flow is in a stationary state. Normally a tumor would grow further during this time frame. However since we do not model pharmacodynamics (i.e. cell killing) it seems reasonable to make this simplification. Parameters for our base case are derived from data for Doxorubicin, a commonly used chemotherapeutic drug. assume that initially the tissue is “clean” i.e. without drug. A bolus injection into the hosts body is modeled by the time varying blood plasma concentration 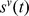. For an injection we take the exponential function 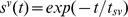 with the time scale 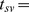 1 h. Our results are presented with unit-less normalized concentrations for which 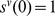.

The distribution of Doxorubicin is usually observed in vivo with the aid of fluorescence imaging, e.g. in [43], [44], [48]. Typically one observes exponentially decaying concentration profiles around tumor vessels, at least during the first few hours. Eventually eventually drug is distributed relatively evenly. When the blood is cleared of drug, molecules diffuse back into the blood stream and so the concentration near vessels decreases again. The overall drug level decreases over the course of a few days until all drug is cleared from the tissue.

**Figure 7:**
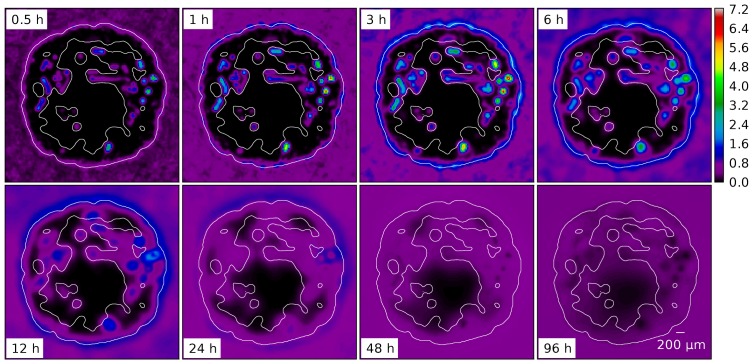
Series of snapshots of the rug distribution 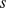 of a typical base case system. Times and length scale are as indicated. The contour delineates the viable tumor mass.

Our simulations results agree well with this observation. To illustrate this, Figure 7 shows the spatiotemporal evolution of the concentration 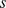 in a sequence of snapshots. The data are taken from one of the 15 systems we considered as the base case. See Supplement S16 for a video.

Upon closer inspection of these figures and comparison with Figure 4 A it becomes apparent that some vessels are not releasing drug. The explanation is that close to the tumor rim we have two classes of vessels with comparable radii but different permeabilities: (i) Mature vessels stemming from arterioles or venules which release little drug and (ii) dilated capillaries. Toward the tumor center these differences vanish due to wall degeneration.

**Figure 8:**
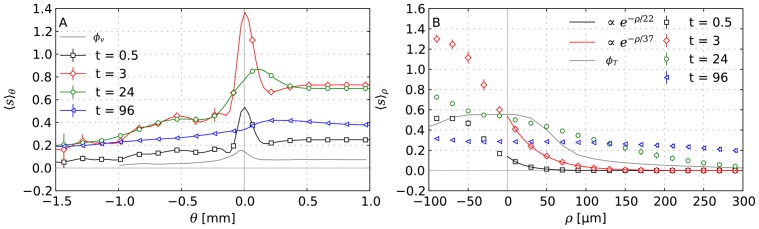
Profiles of drug concentrations at different times. (A) radial distributions of the drug concentration vs. 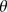 similar to Figure 5. (B) the same concentration distributions are plotted vs. the distance 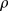 from nearby vessels. 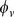 and 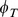 show the profiles of the volume fractions of vessels and tumor cells, respectively. Early on, the concentration plots in (B) can be fitted with an exponential in agreement with experimental data as given in the legend. Each data point corresponds to the average over a small 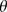 or 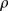-interval, where 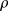 is the distance from vascularized regions similar to 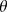 (see text). The error bars indicate the standard deviation of the ensemble.

In Figure 8A drug concentrations are plotted as average 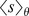 against 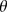 in the same way as Figure 3, i.e. as average over shells of constant 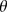. Initially these drug profiles show a strong similarity with the profiles of the vessel volume fraction which is also plotted. Both have a peak at the tumor rim and decay into the tumor to significantly lower levels than in normal tissue. Of course, we expected such a correlation, because the “bulk” transvascular flux is proportional to the vessel surface area. This proportionality also implies a faster drug uptake by diffusion once blood is cleared from drug. Therefore one might naively expect that the tumor rim is cleared fastest of drug, afterwards normal tissue, and finally the tumor center. The actual result at t = 96 h is different, namely that the profile is relatively flat and monotonously increasing into normal tissue. Following the discussion below, it is clear that convection fulfills a significant contribution to transport, moving drug outwards and flattening the profile. This is studied in more detail considering a diffusion dominated system in the the case (iii) (see below).

The ratio of convection to diffusion is quantifiable by the Peclet number which is defined as 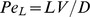, where 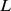 is a characteristic length, 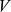 the velocity and 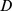 the diffusion constant. 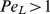 means that transport is convection dominated and 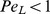 means diffusion domination. It depends on 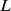, so we analyze our system by determining 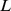 while requiring that 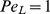 whereby we denote respective 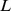 as 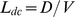. The mean over the tumor 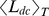 is 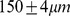. This practically the same length as the diffusion range of oxygen, i.e. the distance from drug sources (vessels) up to which viable tumor cells are present. Hence diffusion and convection are predicted to be equally relevant. In contrast 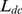 is two orders of magnitude larger within normal tissue which means that transport to spaces in-between capillaries is strongly diffusion dominated (see Supplement S13 Figure 1A for a spatial 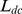 map).

In Figure 8B the mean drug concentration 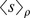 is shown against the distance from tumor vessels 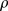. The profiles were generated exactly like the plots against 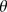 by defining 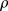 as the (signed) distance from the region where the vessel volume fraction 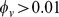 which captures all vessels. Another interpretation of 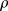 is a penetration depth into the cuffs surrounding vessels and further into necrotic regions. It shows that over the first few hours 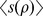 is very well fitted by an exponential decay function 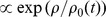. Where the length scale 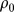 is approximately 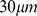, which is in good agreement with [43], [44], [48]. During the 3 to 24 hours period the exponential behavior vanishes. Thereby the level at the “tail” increases while the level in vascularized regions decrease. After 48 hours the profile is almost flat within the local fluctuations at about 30% of the peak value at 30 min.

For the further analysis and quantification of drug delivery we have to introduce an appropriate metric that represents the time-independent spatial distribution of drug doses. Quantities which commonly enter pharmocodynamical models are the maximal concentration over time and the area under curve (AUC) which is the time integral of the concentration. Even in the case of Doxorubicin, which has been known for a long time, models using either one have emerged, see e.g. the discussion in [49]. Mathematically those quantities correspond to the 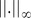 and 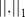 norms (over time), respectively. 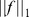 can be bounded by 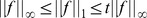 where 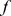 is a proper function and 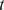 is the observation duration. This bound is of course not very strict so it seems justified to consider both. We use intracellular concentrations since we assume that drug has to enter the cell to e.g. bind to DNA in order to efficacious. Our denotations are 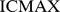 for 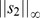 and 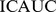 for 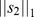.

**Figure 9:**
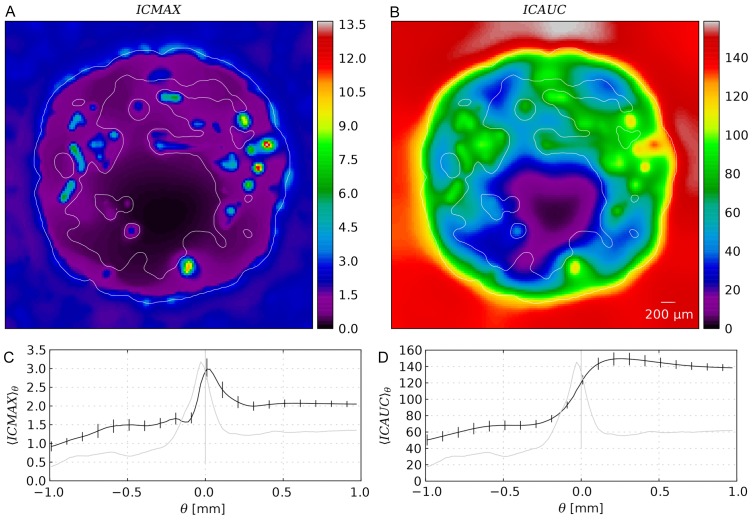
Spatial distributions of drug exposure metrics. (A) Snapshot of the intracellular maximal concentration 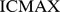, and (B) snapshot of the time integrated concentration 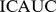 from a typical simulation result from the base case. The contours delineate the viable tumor mass. (C) and (D) Plots of 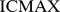 and 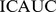 from the base case as ensemble averaged (15 systems) radial distributions (black) similar to Figure 5. In addition, the vessel volume fraction is plotted as well (grey).

As expected we find the highest values near tumor vessels from where the it decreases into the unvascularized (necrotic) regions further away, see Figure 9A and B. 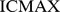 and 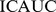 are qualitatively similar and also display strong similarities with early concentration distributions at ca. t  = 3 h. 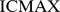 is comparably sharp and in fact approximately 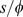 at t  = 3 h. The extracellularly dissolved drug is negligible since its concentration is two orders of magnitude lower. Hence, 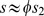. Obviously 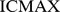 is very sensitive to the high initial drug levels, whereas 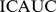 gives more weight to later smoother distributions and thus appears more blurry. Their penetration depth is about 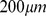 so the whole viable tumor mass obtains significant (meaning non-zero) contributions. The ratio of local maximum to minimum for the AUC is 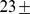 whereas it is 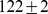 for 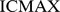. In Figure 9C and D, ensemble averages of 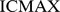 and 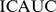 are plotted as radial distributions together with the vessel volume fraction. The correlations are obvious and can be explained by the same reasoning as for the time-dependent concentration distributions. The displayed compartmentalization in decreasing exposure towards the center and peripheral peak is qualitatively retained for most of the parameter variations except for extreme cases with drastically increased interstitial transport rates such as case (v) (see below).

### Variations

In this section we discuss a number of physiologically relevant variations of the base case scenario. We consider the following cases separately:

- Heavier drug particles.
- Prolonged infusions.
- Neglected Convection.
- Vascular permeability.
- Hydraulic conductivity of interstitium.
- Amount of normal lymphatics.
- Tumor lymphatics.

For all cases we produced the corresponding data shown in Figures 3, 4, 5, 6, 7, 8, and 9. They are compiled in Supplements S2, S3, S4, S5, S6, S7, S8, S9, S10, S11, and S12. Videos visualizing the spatiotemporal evolution of the concentration distributions in cases (i), (ii) and (iii) are provided as Supplements S17, S18 and S19.

**Figure 10:**
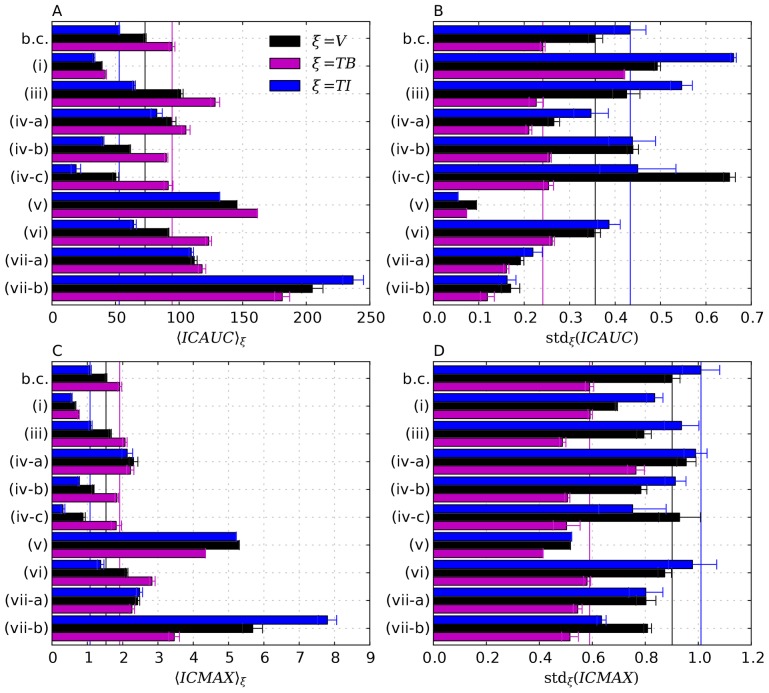
Comparison of the means and STDs of the probability distribution functions (PDFs) of 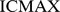 and 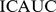. These quantities are presented for different regions, where each region is associated with its own PDF using 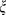 as index, where 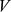 stands for the viable tumor mass, 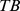 for tumor boundary and 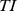 for tumor interior (see text). Spatial mean, indicated by brackets (or STD, indicated by 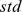) are first computed for all systems in the ensemble and then averaging over the ensemble. The error bars show respective deviations from the average within the ensemble. The meaning of the case labels is as follows: (b.c.) base case, (i) heavier drug particles, i.e. molecular mass increased by a factor of 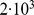. (iii) neglected interstitial convection, (iv) tumor vascular permeability scaled by a factor of 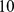 (a), 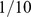 (b), and 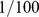 (c), (v) hydraulic conductivity of interstitium scaled by a factor of 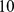, (vi) amount of normal lymphatics scaled by a factor of 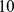, (vii) presence of 10% (a) and 100% of the normal lymphatics amount in the tumor. Note that (ii) (prolonged infusions) are not shown due to the scale.

Below we discuss the physiological implications of these results. In Figure 10 we present a comparison of the mean 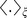 and standard deviation (STD) 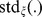 of the 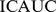 and 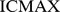 distributions. 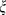 stands for a region over which mean and STD are taken. 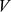 denotes the viable tumor where 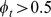, 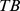 the boundary where 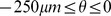 and 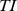 the interior where 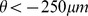. This serves as an assessment of drug delivery efficiency. Higher mean, and smaller STD (i.e. less spatial fluctuations) means better delivery. Histograms representing actual probability density functions for 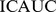 and 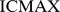 are shown in comparison in Supplement S13.

#### (i) Heavier drug particles

In this case we consider a drug with a molecular weight of 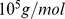. This corresponds to the application of viruses or nano particles as delivery system, which we realize by the adjustment of diffusion related parameters, namely 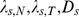 and 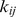. For a particle performing Brownian motion, the diffusion constant scales with 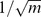 of the particle mass 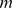. Hence we scale those parameters by 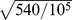, where 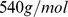 is the molecular weight of Doxorubicin from the base case. This leads to a strongly convection dominated transport where 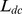 is of the order of 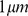.

**Figure 11:**
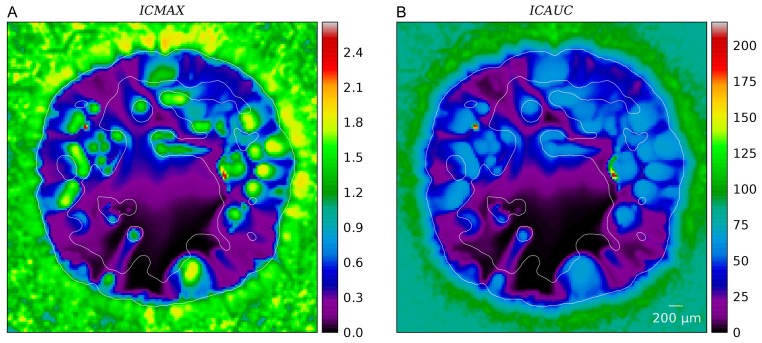
Spatial distributions of (A) 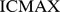 and (B) 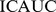 for the case (i). Here 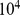 times heavier drug particles are considered which renders diffusive transport insignificant (see text). The presentation is analogous to Figure 9 of the base case.

We see that for heavy particles the IF flow clearly dominates the drug distribution as seen in Figure 11 (see also Supplement S2, and the video provided in Supplement S17). As a result the tumor contains many small, isolated regions where a small amount of drug is delivered. This is reflected by the PDFs (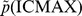, 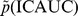) which are broader and lower values are more common. Interestingly, the 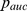 distribution completely changes from bell shaped to almost box shaped, see Supplement S13 Figure 2. The mean values 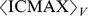 and 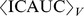 decrease by ca. 50% which can be expected due to the decreased transvascular diffusivity.

#### (ii) Prolonged infusions

Chemotherapy often follows a complicated schedule with several prolonged infusions, which is important to avoid toxicity to normal tissue. The basic idea is that drug given in low concentrations accumulates in the tumor, whereas the concentration in normal tissue remains tolerable. As a simple case we consider the administration of a prolonged infusion. In (ii)a during a 24 h period and in (ii)b continuously during 96 h. The 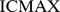 and 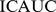 distributions, are surprisingly similar to the base case (see Supplements S3, S4 and the video Supplement S18). The difference lies in the scale. The average concentration 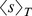 increases approximately linearly in time for as long as the infusion is active.

That means even by the end of our simulation at t  = 96 h the tumor is far from saturated. We expect this to occur when influx equals removal of drug. Assuming that 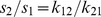 in a quasi steady state the time scale for a purely diffusing particle to move 1 mm can be estimated via the diffusion law 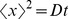 as 1700 h, which is a reasonable estimate for the saturation time scale. Note that our results likely overestimate concentrations and underestimate the speed of the transport due to the lack of saturable cellular components.

#### (iii) Neglected Convection

**Figure 12:**
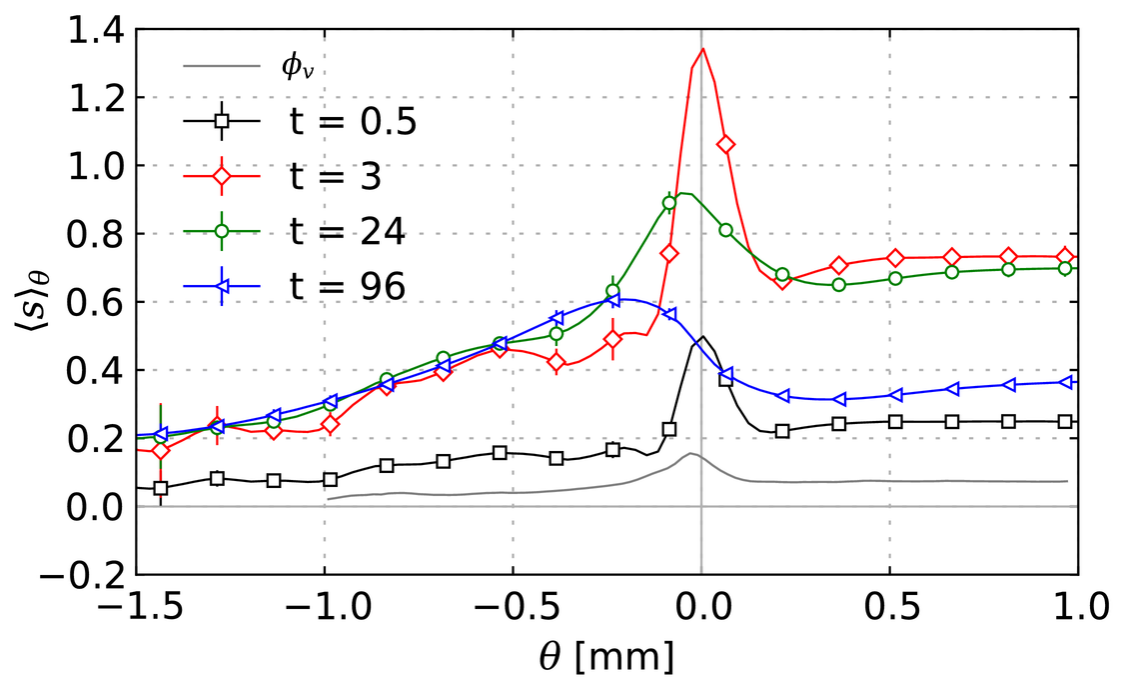
Drug concentrations in the case (vii) where convective transport was neglected. Results are presented as averaged profiles plotted vs. 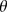 as in Figure 8.

This case serves to gain insight in the role of convective transport of drug through the interstitium. For this purpose the convective term in (20) was neglect. Figure 12 shows that initially (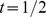 and 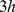) this case is indistinguishable from the base case. But later, 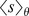 profiles remain peaked within the tumor, leading to significantly increased peripheral drug concentrations. In fact the average concentration in the center remains nearly constant up to a ca. 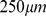 wide peripheral shell (see also Supplement S5, and the video Supplement S19). Thus, convection has the effect of “flattening” the profile apparently by driving additional drug drug into the neovascularized rim where it is reabsorbed once the blood stream is cleared of drug.

**Figure 13:**
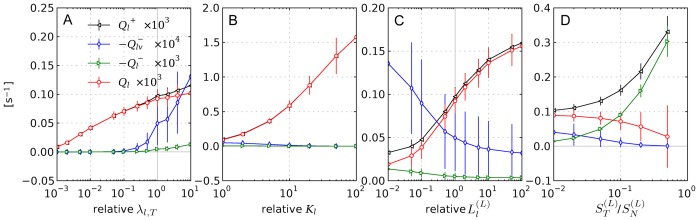
Mean fluid source rates over the whole tumor in dependence on varying parameters. 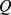
 with its sub and super scripts refer to contributions to the source term as defined in (12), (23). The plotted mean values are defined simply as ensemble averages of the spatial means over the tumor. The error bars show the standard deviations within the ensemble. The subplots depict the following cases: (A) Variation of the upper vessel wall permeability bound 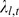 (case (iv)). (B) Variation of the interstitial permeability coefficient 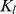 (case (v)). (C) Variation of the lymphatic wall permeability in normal tissue (case (vi)). (D) Variation of the lymphatic density in the tumor given as fraction of the normal tissue lymphatic density 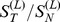 (case (vii)). Except for in (D) the ordinate scale is relative to the base case.

In the following cases (vi), (v), (iv) and (vii) we analyze the effect of varying permeabilities. In [3] it was shown that the IFP profile depends only on the ratio of vascular to interstitial hydraulic conductivity. Here we have spatially varying coefficients but the same scaling law is expected. Nonetheless we vary both parameters since their effect on the velocity and thus drug transport is different. Beside the IFP it seems appropriate to consider the actual flow through the tumor. We quantify this by the mean values 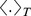 of the following components of the source term in (12) : 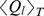 measures the amount of fluid that must leave the tumor through its boundary in interstitial space. 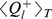 is the amount of extravasated fluid since vessels are the only sources. 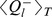 is the amount of fluid taken up by vessels or lymphatics. Note that 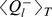 is typically an order of magnitude less than 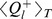, which is clear since there should be very little back flow into vessels. Their response to parameter variations is shown in Figure 13.

#### (iv) Vascular permeability

We vary 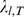 between 1/1000 and 10 times the b.c. value for leaky tumor vessels. Note that this does not simply scale all permeabilities (i.e. 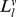) equally, rather 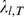 is the cutoff for 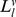 which increases up to this value for 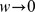 (see (15)). Also note the conductivity of normal capillaries is 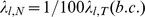.

**Figure 14:**
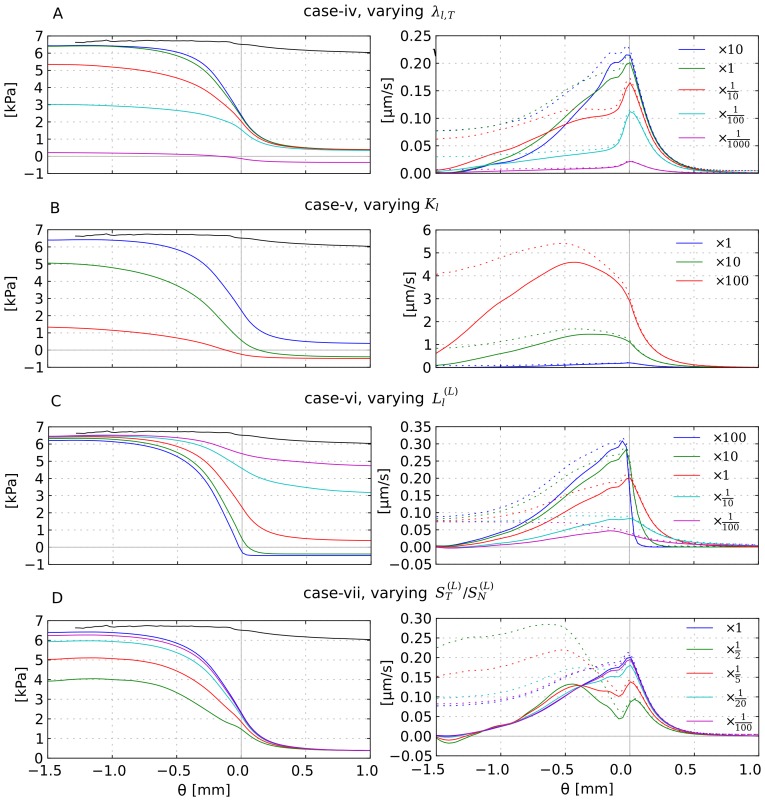
Radial distributions as result of parameter variations. Left: IFP, right: IFF, averaged over 15 system analogous to Figure 5. The relative deviation from the original base case parameter values is given in the figure legends, except in (D). The considered cases are as indicated in the sub-figure heading: (A) Variation of the upper vessel wall permeability bound 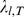 (case (iv)). (B) Variation of the interstitial permeability coefficient 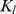 (case (v)). (C) Variation of the lymphatic wall permeability 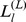 in normal tissue (case (vi)). (D) Variation of the amount of tumor lymphatics 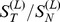, where the legend shows 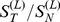 directly (case (vii).

Increasing 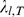 has little effect on the IFP and IFV profile since the IFP already approaches rapidly the blood pressure, see Figure 14 A. The plots of the source terms (Figure 13 A show that for increasing permeabilities most of the little additional flow is reabsorbed into vessels (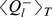). The flow that crosses the boundary is insignificantly altered. For lower permeabilities the uptake 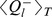 decreases much more rapidly than the influx 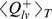. The latter varies weakly within one order of magnitude over the whole 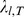 range, implying that the hydraulic resistance of other components, i.e. interstitium and lymphatics limits the flow. Interestingly, setting 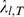 to the level of normal vessels is not enough to lower the IFP to a normal level. It is only reduced by about 50%. Further reduction to 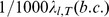 yields near zero IFP (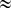 0.1 kPa) but an outward gradient persists.

Predictions for drug delivery were made for 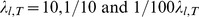 of the base case. See Figure 10 (iv)-A to C (see also Supplements S6, S7 and S8, respectively). In conjunction with 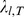 we also varied the diffusive permeability 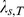 by the same factor. The mean 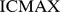 and 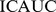 level in 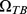 are invariant since this regions exhibits rather normal vessels since leakiness increases gradually toward the center. In the interior, 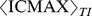 and 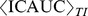 increase with the permeability as expected. Here too, for (iv)-A we find 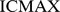 levels in the tumor which are comparable to normal tissue.

#### (v) Hydraulic conductivity of interstitium

The base case considers a dense tissue which implies a low conductivity due to the dependence on the available interstitial volume. Sparser tissues were estimated to be orders of magnitude more permeable [50]. To analyze such a situation we scale the tissue permeability coefficient 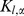 up to 100 times simultaneously for 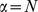 and 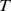. For brevity, 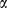 is omitted in the following. Increasing 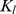 alone produces unrealistic results where the IFP within normal tissue rises well above 0. Hence the lymphatic permeability 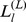, was also increased by the same factor.

With increasing 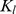 we observe a decreasing IFP which drops to ca. 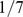 of the base case value for a 100 times increase of 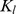, as shown in Figure 14B. The IFV decreases sub-proportionally, up to a factor of 20. Interestingly, the region where most fluid is extravasated shifts from close to the boundary to the tumor center. Concomitantly one finds a shift of the peak in the 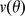 toward the tumor center and less back flow through vessels as compared to the base case as indicated by lowered 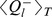. For the total flow 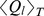 we can identify two regimes: Up to a 10 times increase we see an approximately linear variation, and for larger 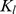 a logarithmic behavior.

Drug delivery is analyzed for an increase of 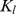 by a factor of 10, where we additionally upscale the diffusion constant 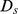 by the same amount (see Supplement S9 for additional figures). This is rather ad-hoc but based on the assumption that the free volume fraction and the amount of ECM components varies, on which the effective diffusion constant and IF permeability linearly depend on as a first order approximation. As a result we obtain a significantly more homogeneous 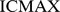 and 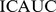 distributions. See the comparison Figure 10B,C-(v) vs. (b.c.). The effect is most drastic for 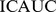 but also the 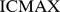 fluctuations over 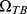 are significantly reduced. Remarkably, the tumor interior now shows higher drug concentrations (Figure 10A-(v)) than the exterior. The fact that the drug delivery to the interior is comparable to normal tissue is surprising since permeabilities were adjusted also for normal tissue not just the tumor.

#### (vi) Amount of normal lymphatics

The lymphatic system is not well documented due to the lack of specific markers for its channel walls. In our parameter determination we assumed that it has a similar capacity as the capillary bed. Moreover tumors can induce lymph angiogenesis similar to normal angiogenesis [9] thereby increasing the amount of lymphatics nearby. Hence we consider variations of the source term coefficient 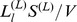 from 100 to 1/100 times the base case (b.c.) value.

As discussed above, the IFP has a “penetration depth” across the tumor boundary which is proportional to 
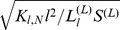
 and indeed we see a variation of this magnitude in the radial profile. Moreover with increasing 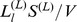 the IFP drops asymptotically to the lymphatic pressure 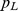, which can be explained by the analogy of an electrical resistor. A 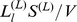 decrease on the other hand drives the IFP in unrealistically high. The central tumor IFP is thereby relatively invariant to these parameter changes, see Figure 14C. The IF velocity varies according to the gradient across the boundary and can be increased by up to 50% in the extreme case. The global flow 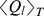 increases by about the same magnitude. The average back flow 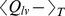 decreases insignificantly within the error bars. On the other hand for low 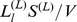 more back flow is observed, which is reasonable since the uptake capacity of lymphatics is decreased.

For drug transport we only consider the case with a factor of 10. Qualitatively the 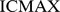 and 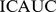 distributions appear similar to the base case (figures are provided in Supplement S10). The increased flow apparently leads to higher drug levels all over the tumor, but the effect is strongest in the 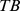 region. Spatial fluctuations are also insignificantly changed. Thus we can conclude that the delivery slightly improved. Given that the fluid transport rate 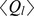 increased by 50% this is surprising since case (i) demonstrated that a larger convection-diffusion ratio can have a negative effect.

#### (vii) Tumor lymphatics

In this hypothetical case we assume that functional lymphatics exist within the tumor. We model this by a non-zero lymphatic sink coefficient 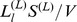 as a fraction of the coefficient in normal tissue. This differs from taking a higher tissue conductivity by the assumption of the underlying channel organization. The latter case implies a grid like structure whereas the former implies a hierarchical structure where exchange takes place over the thinnest channels analogously to blood vessel network. Those lymphatic capillaries can be expected to exhibit approximately equal blood pressure over the whole tissue. Hence the use of homogeneously distributed sinks with lymphatic pressure 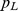.

Resulting IFPs and IFV plots are shown in Figure 14D. As can be seen an increasing presence of lymphatics “pulls” down the IFP to 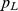. The outward velocity component 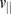 decreases proportionally as can be expected based on the IFP gradient. But the actual velocity magnitude 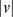 increases with the amount of lymphatics. This can be expected as well since the velocity field is increasingly superimposed by flow away from vessels to nearby locations where fluid is absorbed. This is also reflected by increasing transvascular flow 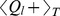, decreased flow across the boundary, 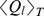 and the back flow 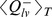 as shown in Figure 13C.

We computed the drug transport for systems with (a) 0.1 and (b) 1 times the normal lymphatics (see Supplements S11 and S12, respectively). What we would expect is that the changed flow patterns (directed away from vessels) facilitate the delivery to more distant regions. As Figure 10A and B-(vii) show this is indeed the case. Even in (a) a significant improvement is achieved. The 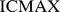 and 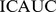 levels in the rather unrealistic case (b) are increased dramatically. This must also be attributed to leaky tumor vessels. Thereby spatial fluctuations are either unchanged (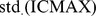) or significantly reduced 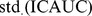.

## Discussion

Based upon an extension of our model for remodeling of tumor vasculature introduced in [15], [16], [18], [19] we studied interstitial fluid flow (IFF) and drug transport.

Since the spatial details of IFF depends crucially the spatial arrangement of blood vessels and their blood pressure we considered, for the first time, a model for IFF and drug transport that involves a realistic arteriovenous initial vascular network evolving dynamically with the tumor. As a result tumor vessels are connected to a wide variety of original host vessels - from capillaries to ca. 50 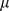
m arterioles and veins. Blood flow is determined for the whole system including the whole remaining host vasculature which comprises not only a single parent vessels but many vessels covering the entire simulation domain. It should be emphasized that an arteriovenous initial vasculature produces blood flow patterns and blood pressure fields that differs substantially from grid-like arrangements [15], [18]: the latter usually have a fixed blood pressure gradient direction imposed upon them which can also (unrealistically) impose a preferred direction on tumor vessel growth and skew the IFP distribution. For a tumor blood vessel network emerging from an arteriovenous initial vaculature we obtain much higher blood ow rates through individual vessels due to arteriovenous shunts and circumferential growth, and more irregular (spatial) blood pressure distributions. Due to transvascular coupling these circumstances are also relevant for the IFP, IFF and drug transport.

A first remarkable result is that in spite of an expected IFP plateau within the tumor IFF does not cease and still allows for substantial convective transport, which is opposite to the currently prevailing view [3]–[9] that states that increased IFP poses a barrier to successful drug delivery within tumors. The physical explanation is simply that it is misleading to consider the pressure drop along the vessel wall alone as the driving force for IFF - in principle the complete network of hydraulic resistors has to be taken into account to obtain reliable predictions. Qualitatively it is sufficient to bear in mind that for IFF the tumor is series of resistors with a fixed potential outside the tumor (the lymphatics of healthy tissue) - decreasing one resistor, for instance by increasing the permeability of the vessel walls, generally *increases* the flow, in spite of the lower potential drop along the decreased resistor.

Another interesting result is that heavy macro-molecules are still distributed more or less evenly into viable areas in the tumor perimeter in spite of a pronounced outward IFP gradient there, which one could naively expect to remove drug before an efficacious dose is achieved. The reason is IFF between tumor internal vessels, that transports macro-molecules convectively from leaky high pressure vessels through the tumor tissue into neighboring low pressure vessels. In the following we discuss our results quantitatively in detail.

It is already established that leaky tumor vessels and lack of tumor lymphatics lead to a drastically increased hydraulic pressure of the interstitial fluid (IFP) in the tumor and that the resulting gradient drives fluid out of the tumor with a velocity of 0.1 to 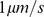 [4], [51] and our results agree with these experimental observations very well. Our model predictions of the central IFP is ca. 6 kPa, which is at the upper limit of experimentally observed values. This value is also higher than in normal capillaries due to the coupling with higher level arterioles in which the blood pressure is naturally higher. The vessel walls of arteries and veins within the tumor become leaky [21] and thus increase their conductivity, which means tighter coupling between IFP and blood pressure which as a result causes the IFP to approach the level of nearby arteries or veins. Indeed, the predicted mean pressure difference between blood and interstitium vanishes towards the tumor center. Local fluctuations produce flow into, out of and in-between vessels which is not necessarily directed outwards. The outward component of the velocity vector dominates in the tumor periphery. Around the tumor perimeter we find a thin layer where where the fluid is absorbed into lymphatics. Absorption into blood vessels frequently occurs deep inside the tumor. It should be noted that the fluctuations among samples are very large, about 100%. Consequently, depending on their location in the microenvironment, seemingly by chance some tumors could be more likely to metastasize through the blood stream than others of the same kind. Although the transvascular flow of tumor vessels is elevated by an order of magnitude compared to normal capillaries, we estimated that only a small fraction of the order of 0.01 percent of the blood flow which enters the tumor is lost into the interstitium. The biophysical factors upon this ratio depends are the vascular morphology and the permeability of vessel walls, interstitium, and lymphatics. Here, dilated vessels and arteriovenous shunts lead generally to elevated flow rates (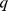) in tumor vessels. Note that 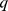 depends strongly on the vessel radius 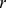, i.e. 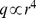.

In addition to the base case we considered scenarios in which the conductivities of vessel walls, lymphatics and the interstitium are varied individually. We observe universally that the influx through vessels, and therewith the IFF through the tumor, increases with the conductivity. The sensitivity to these changes is rather low, even for variations of several orders of magnitude. Lower IFP does thereby not correlate with increased flow. For example the reduction of IFP can be achieved by increasing the tissue conductivity or decreasing the vessel wall conductivity. In the first case the IFF is increased while in the second it is decreased. This relationship between IFF and conductivity can be easily understood on the basis of analogy of our flow equations with an electrical network of ohmic resistors: As an extreme idealization let us consider a linear chain of three resistors, representing the walls of the tumor vasculature, the interstitium and lymphatic walls, whereby the potentials are fixed at the ends. Then of course the current is determined by the total resistance and the voltage drop over one resistor grows with its resistance. An increase of the first resistor (decrease of vessel wall conductance) as well as decrease of the second resistor (increase of tissue conductivity) both imply a lower voltage drop at the second resistor (lower IFP). But the current (the IFF) decreases in the 1st case and increases in the 2nd.

We quantify the local exposure to drug with the help of both the time integrated intracellular concentration (denoted as 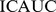 for intracellular area under curve) as well as the maximal concentration (denoted as 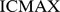 for intracellular maximal concentration), which we consider separately. Our model predicts that on a large scale, drug delivery is compartmentalized similar to the vasculature. Let us subdivide the system into concentric shells with increasing distance from the invasive edge and consider averaged quantities over such shells. Close to the invasive edge we typically find an exposure peak, the location of which is not exactly aligned with the MVD peak due to outward convection. Towards the tumor center the decreasing vessel density leads to a sharp drop down to a plateau at a certain lower bound which is for our base case ca. 50% of the level at the tumor periphery. The height of this plateau depends on various factors including extravasation rate, transport rate through the interstitium, but predominantly vessel density.

The tumor center is threaded by many isolated vessels, and our model predicts exponentially decaying concentration gradients around them in agreement with experimental results e.g. [43], [48]. Over longer time scales of many hours to days, the initial distributions smear out akin to the behavior of freely diffusing particles, and also decrease globally due to reabsorption. The magnitude of these time scales depend on the transport rates, cellular uptake and binding dynamics.

Although we did not study it in detail, we want to stress that cellular uptake and retention dynamics also govern the total amount of extravasated drug to a certain extend because the faster the uptake rate, the lower the interstitial concentration, the larger transvascular gradients which drive diffusive fluxes and counteract re-absorption by convection.

It has been suspected for over a decade that IFF could wash out drug from the tumor. Our model predicts this effect but as drastic in magnitude as expected. Simulation runs in which convective transport was neglected show that a ca. 2.5 times higher drug concentration is retained in the periphery whereas the radial profile of the time-independent exposure measures (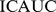, 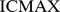) merely exhibit a smoother decay from periphery to center. In the opposite extreme case of non-diffusing particles under normal convection the the interstitial flow causes sufficient flux to achieve significant drug delivery which is on a coarse scale comparable to the base case. Here we do however observe islands in the viable tumor region where no drug is delivered, implying that such tumor fragments could remain viable after treatment. In cases with diffusion, at least small amounts of drug are delivered.

Different tissue types as well as the effect of therapies that improve drug delivery can be described within the model by changing various permeability constants. We varied diffusion constants simultaneously with conductive constants by the same factors as first approximation to hypothetical changes e.g. of the intercellular channel geometry. As a result, global exposure levels and the amount of extravasated fluid correlate well with these permeabilities as well as with each other. Unfortunately relative local fluctuations, i.e. the STD over space of 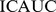 and 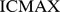 are not correlated with these variations, making them unsuited as basis for achieving are uniform efficacy or dose.

Within the biologically relevant parameter space of which we only considered a small part, analysis of other cases will certainly lead to additional insight. For example in [14] tumor interstitium and capillary permeabilities (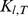 and 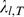 in terms of our model) were varied simultaneously, leading to a flattened tumor IFP profile for increasing permeability. From such observations in an experimental setting one could draw conclusions about the nature of the tumor tissue.

With this in mind a tumor therapy that comprises a treatment that solely reduces the vessel leakiness appears not to be effective. A currently frequently discussed alternative is to improve the efficacy of drugs by turning the ill-formed tumor vasculature normal again [52] by pharmacological means during or before a conventional therapy. A step in this direction is obviously to reduce the leakiness of tumor vessels. However, our simulations predict an actual reduction of the global 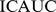 and 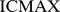 levels while their relative fluctuations may even increase. On the other hand increased permeability leads to significantly improved delivery in the tumor center. Unfortunately this case is unlikely to be a therapeutic goal since it could increase the direct exposure of the blood stream to tumor cells increasing the chance of tumor cells entering it. A good treatment strategy would be to prioritize the maintenance, or fabrication of a dense tumor vasculature, rather than exclusively tightening the leaks in the already sparse vasculature. If a tumor is detected early enough the vasculature could thus be kept intact and deliver drugs effectively, simply due to the amount of functional capillaries, even though their walls are expected to conduct drug worse than leaky walls.

Vascular targeting therapies that take the opposite direction namely that aim to destroy the remaining tumor vasculature completely exists hand have proven to be promising, see e.g. [53]. A common problem in therapy is that tumor cell in vivo are more resistant to treatment than cell cultures. Many factors are involved but not all are purely of genetic nature. For example the lack of oxygen plays an important role for the development of radiation resistance. Or the drug delivery through the sparse tumor vasculature is insufficient, which is supported by our model prediction that the drug concentrations in the tumor are much lower than in normal tissue. If cells in the interior were killed indirectly by vascular targeting, a viable shell around the boundary would remain which could be effectively treated conventionally due to the better vascularization. It is plausible that the removal of the inner tumor vessels would lead to a reduction of the IFP and thereby lower IFF across the boundary. As our results show, the absence of this peripheral flow can improve the drug exposure of the boundary significantly potentially leading to better treatment in combination with vascular targeting.

Interestingly, the 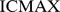 and 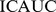 levels increase with the interstitial permeability, lymphatic permeability, or when providing the tumor with a fraction of surviving lymphatics. In addition to that and perhaps more importantly, drug concentrations become more homogeneous as well. This effect is much more prominent for 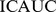 than for 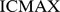, where for the former the spatial STD decreases from over 0.4 to ca. 0.2. With a 10 times increased interstitial conductivity the 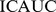 distribution even becomes completely smooth over the whole tumor at levels higher than in normal tissue. Unfortunately, in those cases the IFF is also increased which would likely increase the shedding of TCs into lymphatics, not to mention the danger of having TCs in direct contact with tumor lymphatics. Hence, despite significantly improved delivery the parameters above are certainly not a useful therapeutic target.

The final 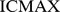 and 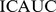 distributions for continuous infusions are hardly distinguishable from the bolus case except for the scale. The reason is that the time scale to achieve a stationary state (estimated ca. 1700 h) is much longer than the infusion periods (24 h and 96 h) which we tried. For Doxorubicin cellular uptake is relatively fast (of the order of minutes) and release is slow (of the order of hours), hence the local concentrations are in a steady state where most of the drug is arrested in cells, lowering the effective transport rates. It can be expected that drugs with low uptake rates perform better in this respect.

## Supporting Information

Supplement S1 **Further details on the model.**
(PDF)

Supplement S2 **Supplemental figures for case i.**
(PDF)

Supplement S3 **Supplemental figures for case ii-a.**
(PDF)

Supplement S4 **Supplemental figures for case ii-b.**
(PDF)

Supplement S5 **Supplemental figures for case iii.**
(PDF)

Supplement S6 **Supplemental figures for case iv-a.**
(PDF)

Supplement S7 **Supplemental figures for case iv-b.**
(PDF)

Supplement S8 **Supplemental figures for case iv-c.**
(PDF)

Supplement S9 **Supplemental figures for case v.**
(PDF)

Supplement S10 **Supplemental figures for case vi.**
(PDF)

Supplement S11 **Supplemental figures for case vii-a.**
(PDF)

Supplement S12 **Supplemental figures for case vii-b.**
(PDF)

Supplement S13 **Supplemental figures.**
(PDF)

Supplement S14 **Video of a growing tumor in silico.** A quarter of the system is cut out to open the view into the interior. The presentation is analogous to Figure 2, i.e. the yellow mass depicts the viable tumor. Void spaces within the tumor are necrotic regions. The blood vessel network is color coded by blood pressure. Red is high (arteries), and blue is low (veins).
(AVI)

Supplement S15
**Video of a growing tumor in silico.** Here we visualized a ca. 200 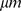 thick slice through the center of the system. The presentation is analogous to Figure 2, i.e. the yellow mass depicts the viable tumor. Void spaces within the tumor are necrotic regions. The blood vessel network is color coded by blood pressure. Red is high (arteries), and blue is low (veins).(AVI)

Supplement S16 **Video of the drug concentration distribution for the base case.**
(AVI)

Supplement S17 **Video of the drug concentration distribution for case i.**
(AVI)

Supplement S18 **Video of the drug concentration distribution for case ii-b.**
(AVI)

Supplement S19 **Video of the drug concentration distribution for case iii.**
(AVI)
