# Correction: Interstitial Fluid Flow and Drug Delivery in Vascularized Tumors: A Computational Model

**DOI:** 10.1371/journal.pone.0110568

**Published:** 2014-10-07

**Authors:** The PLOS ONE Staff

## Notice of Republication

This article was republished on September 25^th^, 2014, to correct an error where incorrect symbols were used in the equations throughout the paper. Please download the PDF again to view the corrected article. The originally published, uncorrected article and the republished, corrected article are provided here for reference.

## Supporting Information

File S1 Originally published, uncorrected article
(PDF)

File S2 Republished, corrected article
(PDF)
