# Supplementary material for: Interstitial Fluid Flow and Drug Delivery in Vascularized Tumors: A Computational Model
Source: PLoS One. 2013 Aug 5;8(8):e70395. doi: 10.1371/journal.pone.0070395 (PMC3734291; doi:10.1371/journal.pone.0070395)
Supplement: Supplement S1 — Further details on the model. (PDF) [file pone.0070395.s001.pdf]

S1 Details of the model

Illustrative figures

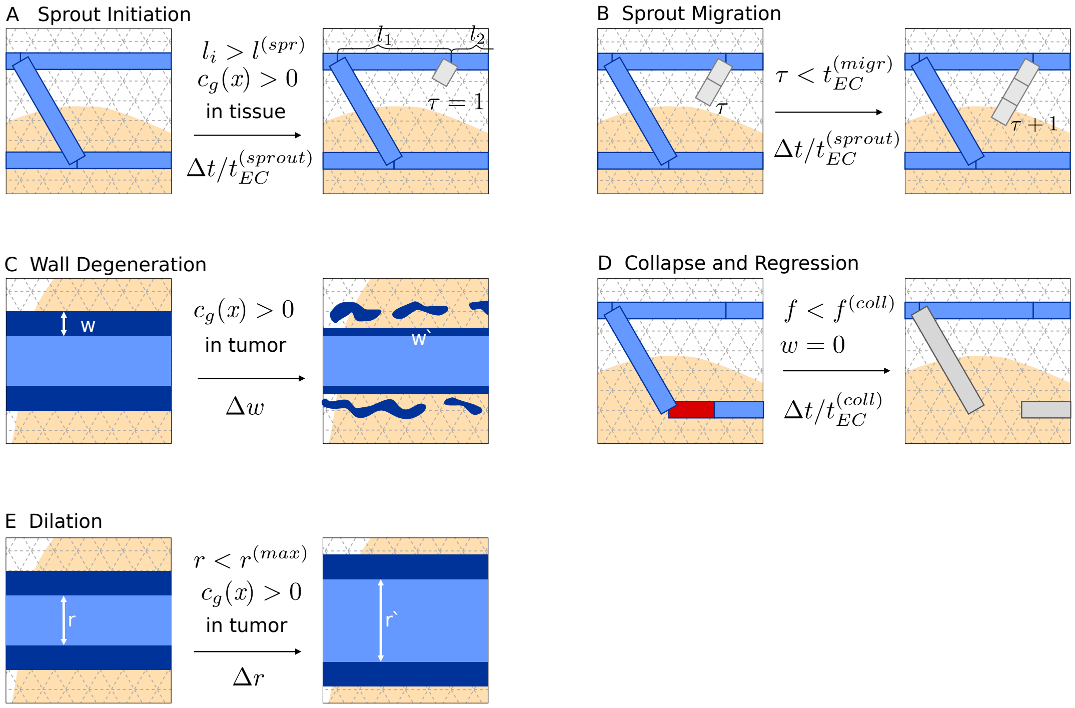

Figure 1: **Illustration of the vessel network remodeling processes.** The contents of the boxes display exemplified states of the vessel network. The vessel segments are shown as blue boxes if perfused with blood, else they are in a gray shade. Dark-blue borders (in C and E) represent vessel walls of varying thickness. The presence of a tumor is hinted at by the light orange region. The state transitions go from left to right as indicated by the arrows whereby the rate parameter is denoted below- and essential preconditions above the arrows, respectively. The note "in tissue" or "in tumor" refers to the location of a segment, see text for the precise definitions these conditions. In (A) a new sprout is generated. In this instance the preexisting segment is split at the branching point. Subsequently a new sprout segment is added with its life-time counter τ initialized to 1. l_i denote the distances to neighboring branching points. Since vessels can be chained together without branching points it is in general required to traverse such chains until $l_i > l^{(spr)}$ or a branching point is found. (B) depicts the further extension of the sprout from (A). It has already been extended to two segments and a third segment is appended. The lengths of sprout segments are always exactly one lattice bond. τ is inherited from the parent segment and incremented by 1 for all sprouts once per remodeling sweep. (C) depicts the degradation of the vessel walls with the rate Δw. Though w is defined as an abstract measure, it can be related to the wall thickness as shown here. w' stands for the value at the next time step, i.e. using Euler's method $w' = w + \Delta t \Delta w$. In (D) an unstable vessel is removed, representing occlusion of blood flow and complete disintegration. Such event is assumed to happen only to vessels with maximally degenerate walls and low wall shear-stresses. Previously attached vessels can become dead ends which means blood cannot circulate through them any more. Such segments are identified by the biconnected component graph-algorithm [1] and excluded from blood flow computations. Since these vessels trivially have $f < f^{(coll)}$, they can collapse easily, resulting in a long ranged effect. (E) depicts the dilation of tumor vessels. The new radius after one time step is $r' = r + \Delta t \Delta r$.

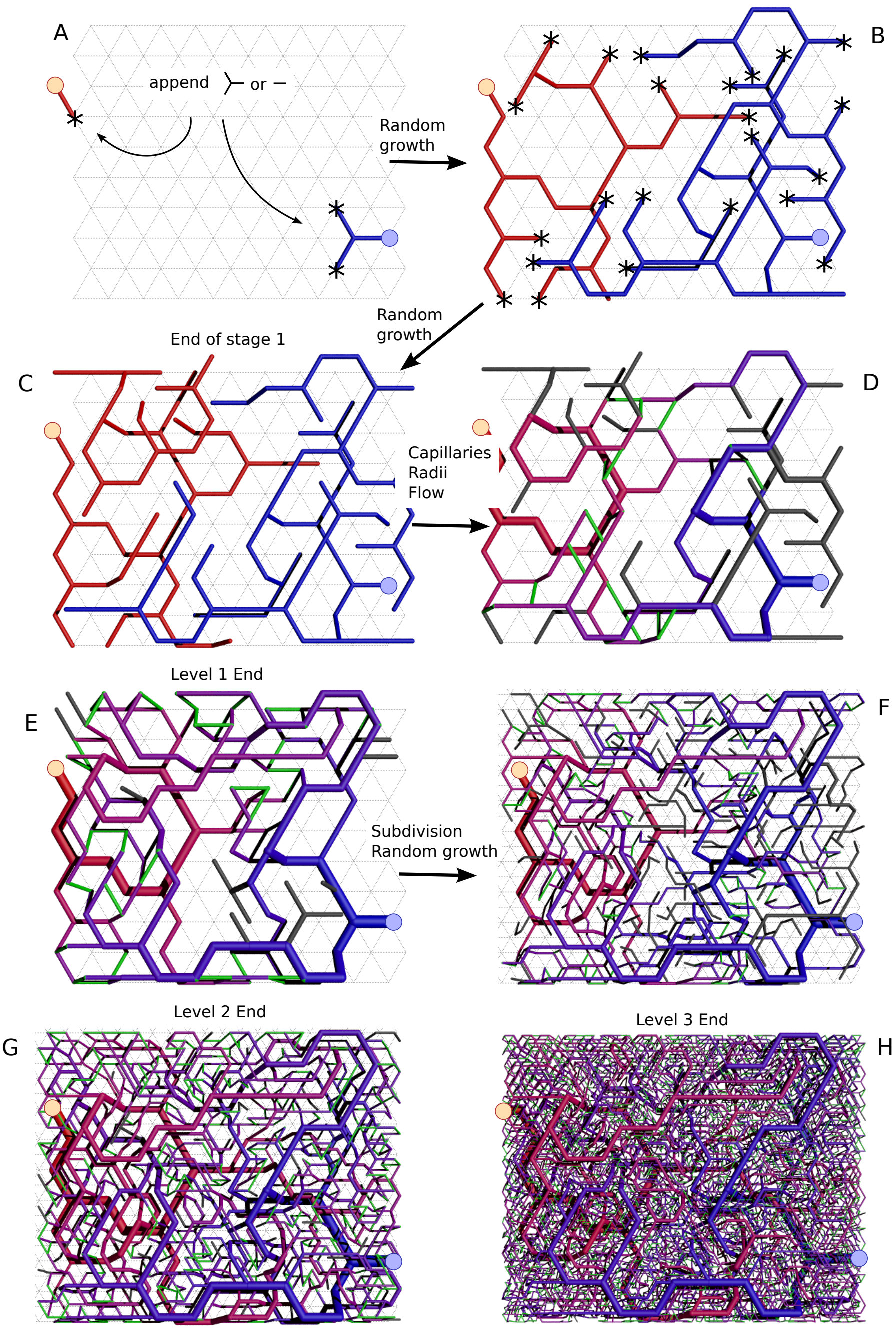

Figure 2: **Sketch of the vessel network generation algorithm.** For illustration we display subsequent actual configurations from a run on a $10 \times 10 \times 2$ lattice. The background mesh depicts the $z = 0$ layer of lattice. The color code for the vessels generally shows arteries in red and veins in blue. If blood flow data is available (D to H) the blood pressure is mapped to a red-blue gradient. The two circles (see A) display the location of the root nodes. Here are just two, but for our other results we placed them in alternating order at all boundary sites of the lattice. (A) shows the state after two growth iterations. Stars indicate the open ends where further elements can be appended (also in B). (B) shows the state during the random growth process. (C) shows the state at the end of the random growth process. Further additions would cause overlap which is forbidden. In (D) capillaries were added, radii computed and blood flow computed. Unperfused branches are displayed in dark gray. Capillaries are green. (E) displays the state after shear-stress based remodeling. (F) shows the network on a lattice which was upscaled once, doubling the number of sites and the length of the original vessel segments to span over two lattice bonds. Previous tips served as starting points for random growth as in A to C. This was followed by determination of radii, generation of capillaries, and computation of blood flow as in C to D. (G) shows the result from (F) after remodeling. Here perfused capillaries are well distributed. (H) shows the final state after an additional up-scaling step.

Initial blood vessel network construction

Random growth: The addition of a new element q at a tree tip is determined by probability weights w_q for all possible additions q. Specifically, we must consider the type and orientation of a new element. Additions resulting in overlap with the existing network are forbidden. Also we allow only additions in the forward direction. For non-admissible configurations we set $w_q = 0$, else we set $w_q = \cos(\varphi)$, where φ denotes the angle between the element and the parent branch. A specific element for addition is then picked according to the probabilities generated from the normalized weights $p_q = w_q/(\sum_q w_q)$.

Remodeling: In addition to the selection of the growth direction we must determine for each site on the network whether a new element is attached, a segment is removed, which is only possible at tree tips or if no changes are made. For these cases the following probability weights for growth w_g, regression, w_d and idleness w_i are given

$$\begin{aligned} w_g &= f/f_{max} \\ w_d &= 1 - f/f_{max} \\ w_i &= \infty \text{ if } f = 0 \text{ and not } N(\boldsymbol{x}), \text{ else } 0, \end{aligned}$$

where N is a boolean indicator function which maps to true if and only if there are perfused vessels within the range of $3\mathbb{L}_h = 240\mu m$, f is the shear stress and f_{max} is the maximal stress over all of the tree tips. The probabilities for an event are given by the normalized weights as above, as well as the probabilities for the element type and its orientation. Against this background we wrote $w_i = \infty$ for the symbolic meaning that it yields $p_g = 0, p_d = 0, p_i = 1$.

Pressure radius relation

The root nodes of the trees have fixed blood pressures as boundary condition. Their value is determined by an empirical formula which was fit to Fig. 5 in [2].

$$\begin{aligned} r' &= r \text{ for a vein, else } -r \\ A1 &= 89, A2 = 18, r_0 = -21, \Delta r = 16 \\ P &= 0.133[A2 + (A1 - A2)/(1 + \exp((r' - r_0)/\Delta r))] \end{aligned} \tag{1}$$

in units of μm for the radius r and kPa for the pressure P. For our typical networks this yields pressures from ca 2.5 to 12 kPa, where the radii of the root vessels are ca. 100 μm.

Radius wall-thickness relation

Initially the vessel wall thickness $w(t = 0) = w^{(init)}$ must be well defined. Guided by [3], we use

$$w^{(init)} = 2r(0.65 - 0.2\log(2r)), \tag{2}$$

depending on the vessel radius r. Sprouts are also created with $w = w^{(init)}$.

Vessel-wall permeability to fluid

In reality vessels have differing degrees of wall permeabilities, e.g. leaky tumor vessels are very permeable, and the thicker the vessel is the less permeable it should be. In order to adequately represent this we utilize the wall thickness property w. The flux across the vessel wall is driven by the pressure difference, where the permeability is the inverse resistance. We assume an ideal system where the resistance of the wall increases linearly with its thickness analogous to an ohmic resistor. This leads us immediately to

$$(L_l^v)^{-1} = \max\left((\lambda_{l,T})^{-1}, \frac{w^v}{w_{(init)}(r = 5\mu m)}(\lambda_{l,N})^{-1}\right)^{-1}, \tag{3}$$

where $\lambda_{l,T}$ and $\lambda_{l,N}$ are experimentally determined permeabilities for capillaries in tumor and normal tissue. $w_{(init)}(r = 5\mu m)$ is initial thickness of capillary walls. Below that, w is a mere abstract representation of the amount of leakiness. Therefore we are free to limit L_l^v from above by $\lambda_{l,T}$ in order to obtain reasonable values for degenerated tumor vessels.

Vessel-wall permeability to nutrients and drug

These permeabilities are denoted L_o^v and L_s^v, respectively. They are defined exactly the same as L_l^v only the indices are o or s instead of l.

Smoothed Heaviside and Dirac Delta functions

We adopt sin/cosine based formulas commonly used in level set and immersed boundary methods, see e.g. [4, 5].

$$\delta_\epsilon(x) = \frac{1}{\epsilon}\begin{cases}\frac{1}{2}(1+\cos(\frac{\pi x}{\epsilon})) & \text{if } |x| \le \epsilon, \\ 0 & \text{otherwise}\end{cases} \tag{4}$$
$$\delta_\epsilon(\boldsymbol{x}) = \delta_\epsilon(\boldsymbol{x}_0)\delta_\epsilon(\boldsymbol{x}_1)\delta_\epsilon(\boldsymbol{x}_2).$$

In addition to the convergence to the "sharp" Dirac delta it has the nice properties that it is continuous and has limited support. The smoothed Heaviside function is the integral over $\delta_\epsilon(x)$ and reads

$$\Theta_\epsilon(x) = \begin{cases}0 & \text{if } x \le -\epsilon, \\ \frac{1}{2}[1+\frac{x}{\epsilon}+\frac{1}{\pi}\sin(\frac{\pi x}{\epsilon})] & \text{if } |x| < \epsilon, \\ 1 & \text{otherwise}\end{cases}.$$

References

[1] Hopcroft J, Tarjan R (1973) Algorithm 447: efficient algorithms for graph manipulation. Commun ACM 16: 372–378.

[2] Pries A, Secomb T, Gaehtgens P (1995) Design principles of vascular beds. Circulation Research 77.

[3] Pries AR, Reglin B, Secomb TW (2005) Remodeling of blood vessels: responses of diameter and wall thickness to hemodynamic and metabolic stimuli. Hypertension 46: 725–731.

[4] Sethian JA (1996) Level Set Methods. Cambridge University Press. URL `http://visinfo.zib.de/EVlib/Show?EVL-1996-149`.

[5] Peskin CS The immersed boundary method. Acta Numerica 11: 479–517.
