# Supplementary material for: Interstitial Fluid Flow and Drug Delivery in Vascularized Tumors: A Computational Model
Source: PLoS One. 2013 Aug 5;8(8):e70395. doi: 10.1371/journal.pone.0070395 (PMC3734291; doi:10.1371/journal.pone.0070395)
Supplement: Supplement S9 — Supplemental figures for case v. (PDF) [file pone.0070395.s009.pdf]

Supplemental figures for case (v)

This SI shows results from simulations where the interstitial conductivity is increased by scaling K_l and D_s by a factor of 10 for tumor and normal tissue.

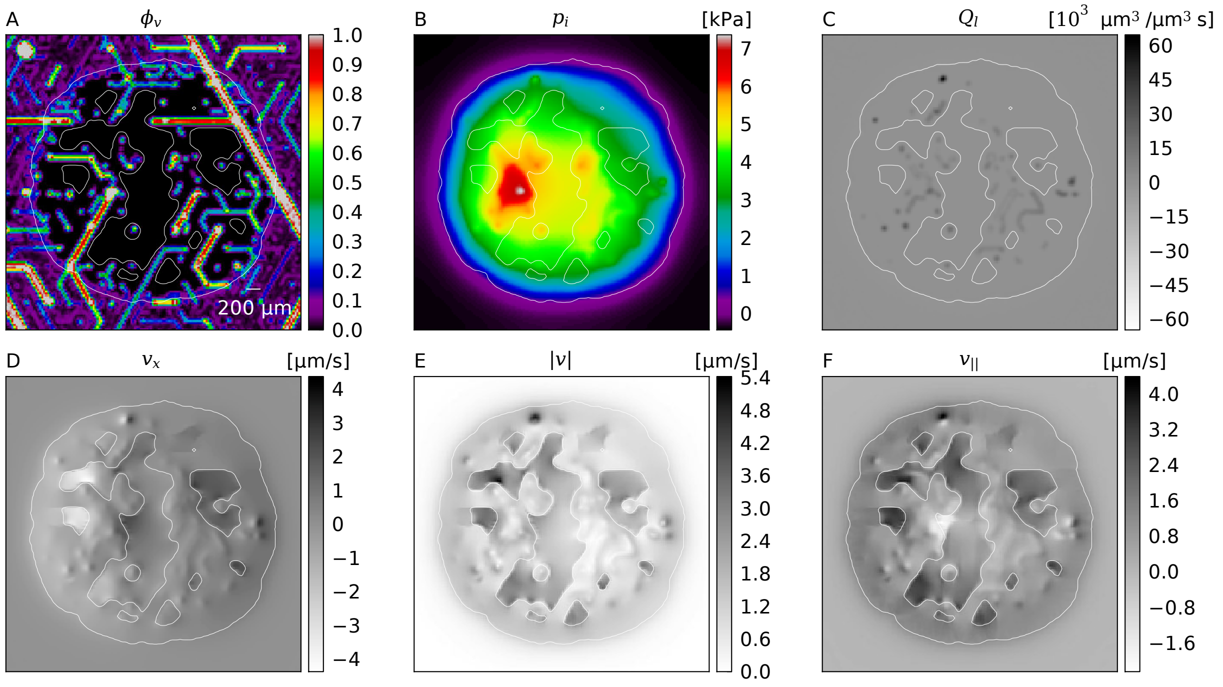

Figure 1: **Snapshots of interstitial fluid flow related quantities.** Corresponds to Figure 4 in the paper.

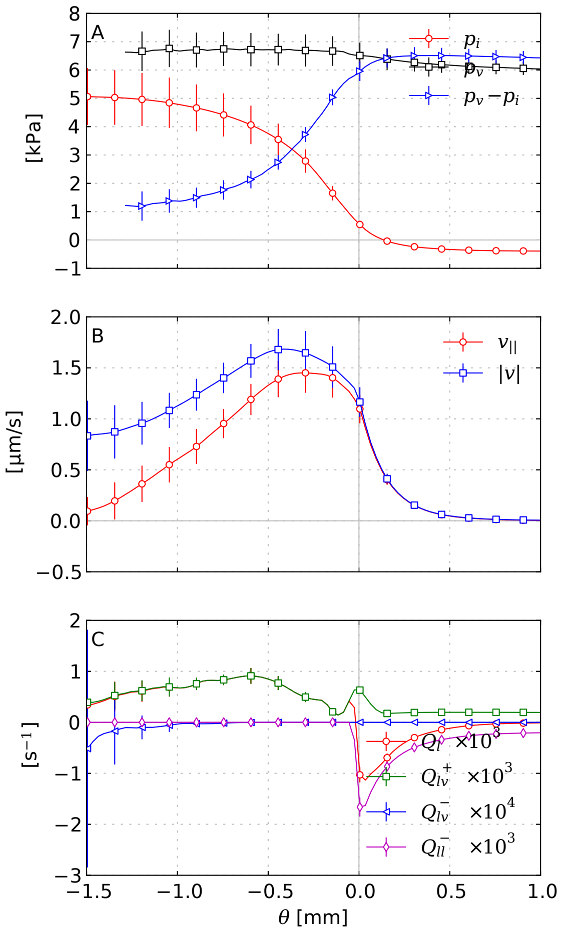

Figure 2: **Averages of IF flow quantities vs. distance from tumor surface θ.** Corresponds to Figure 5 in the paper.

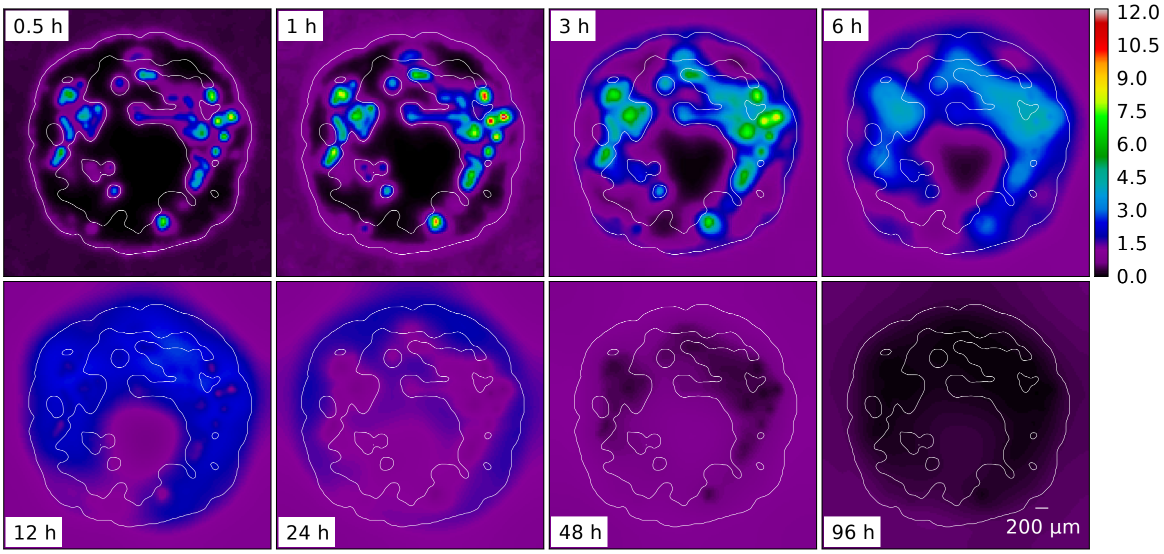

Figure 3: **Drug distribution** s **in a series of snapshots.** Corresponds to Figure 7 in the paper.

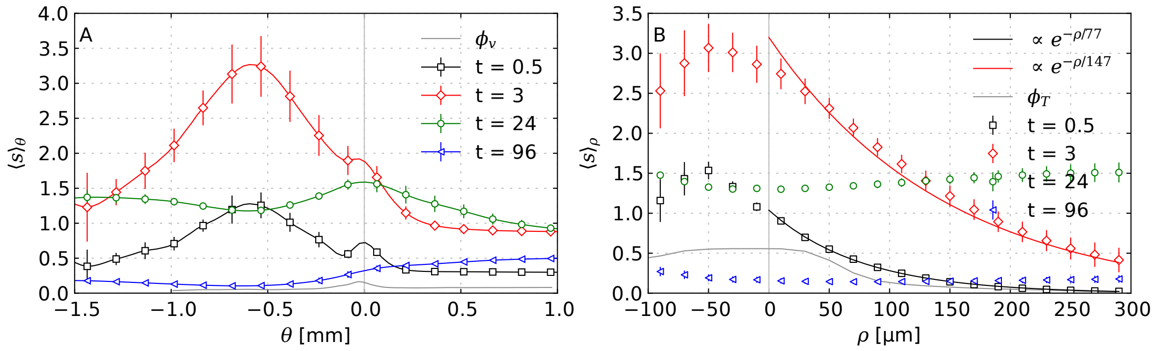

Figure 4: **Drug concentration profiles at different times.** (A) plotted vs. θ, and (B) vs. distance from vessels ρ. Corresponds to Figure 8 in the paper.

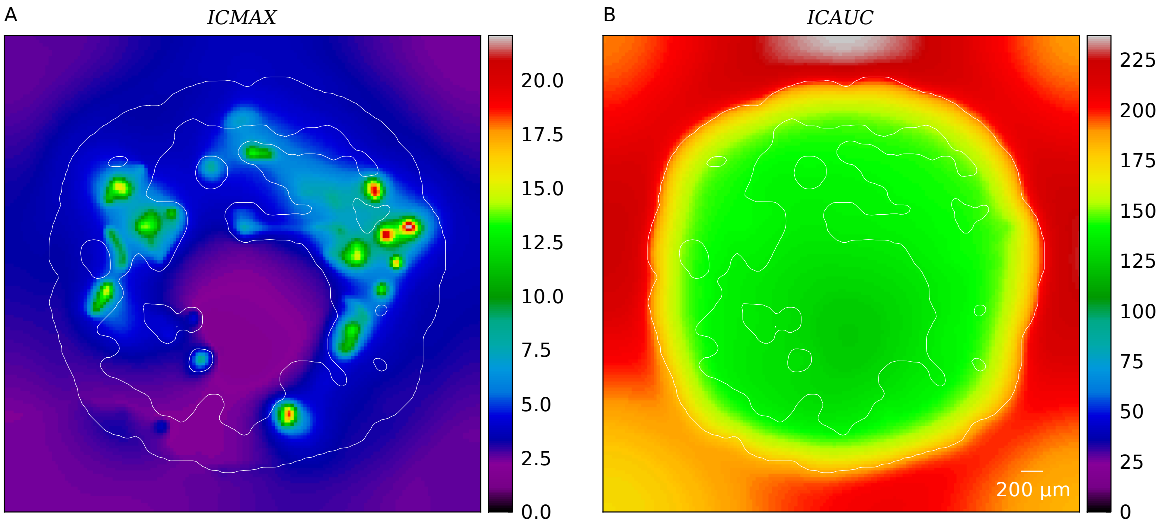

Figure 5: **Spatial distribution of drug exposure metrics.** (A) maximum concentration ICMAX and (B) the AUC ICAUC, taken from a slice through the origin of the system. Corresponds to Figure 9 in the paper.

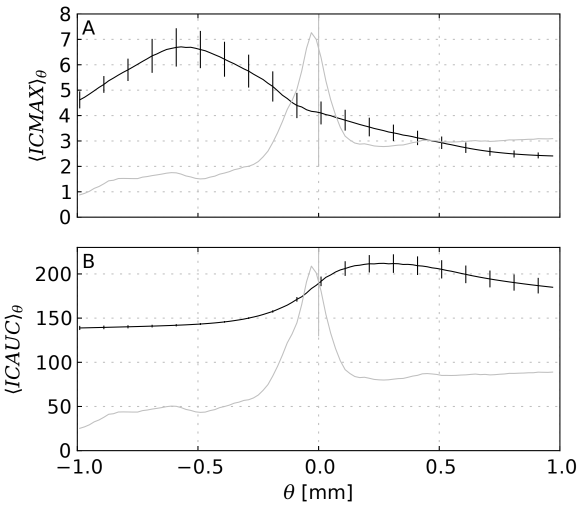

Figure 6: **Drug exposure metrics profiles.** Maximal concentration ICMAX (A) and area under curve ICAUC (B) plotted vs. θ. Corresponds to Figure 10 in the paper.

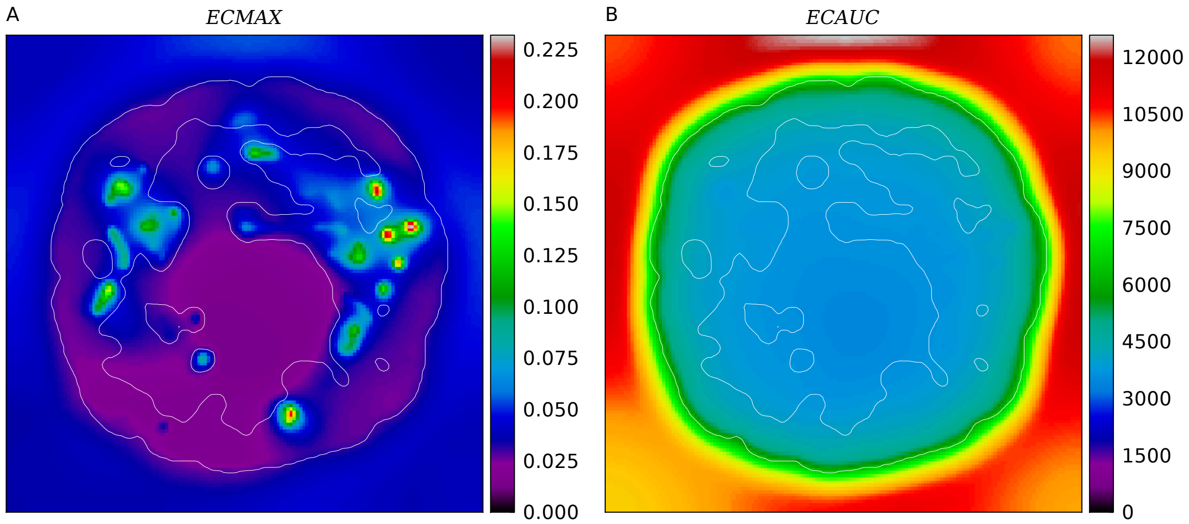

Figure 7: **Maximal concentration (A) and area under curve (B) for the concentration in the interstitial compartment.**

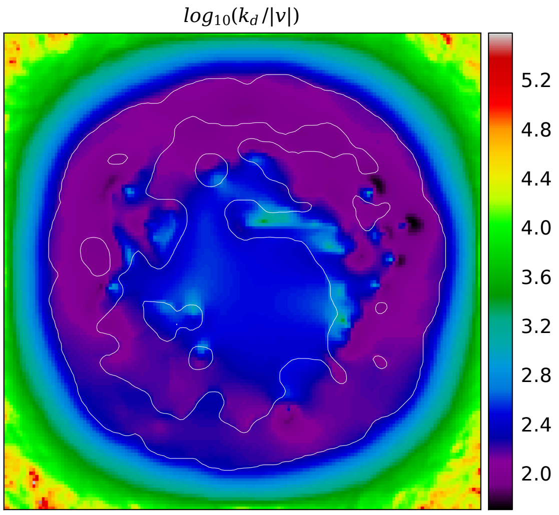

Figure 8: **Logarithmic plot of the length scale L_{dc}.** It is defined by $L_{dc} = k_d/|v|$ following the requirement that the Peclet number equals one, i.e. $1 = Pe = L_{dc}|v|/k_d$. The data is scaled logarithmically.
