# Supplementary material for: Interstitial Fluid Flow and Drug Delivery in Vascularized Tumors: A Computational Model
Source: PLoS One. 2013 Aug 5;8(8):e70395. doi: 10.1371/journal.pone.0070395 (PMC3734291; doi:10.1371/journal.pone.0070395)
Supplement: Supplement S13 — Supplemental figures. (PDF) [file pone.0070395.s013.pdf]

Supplemental figures

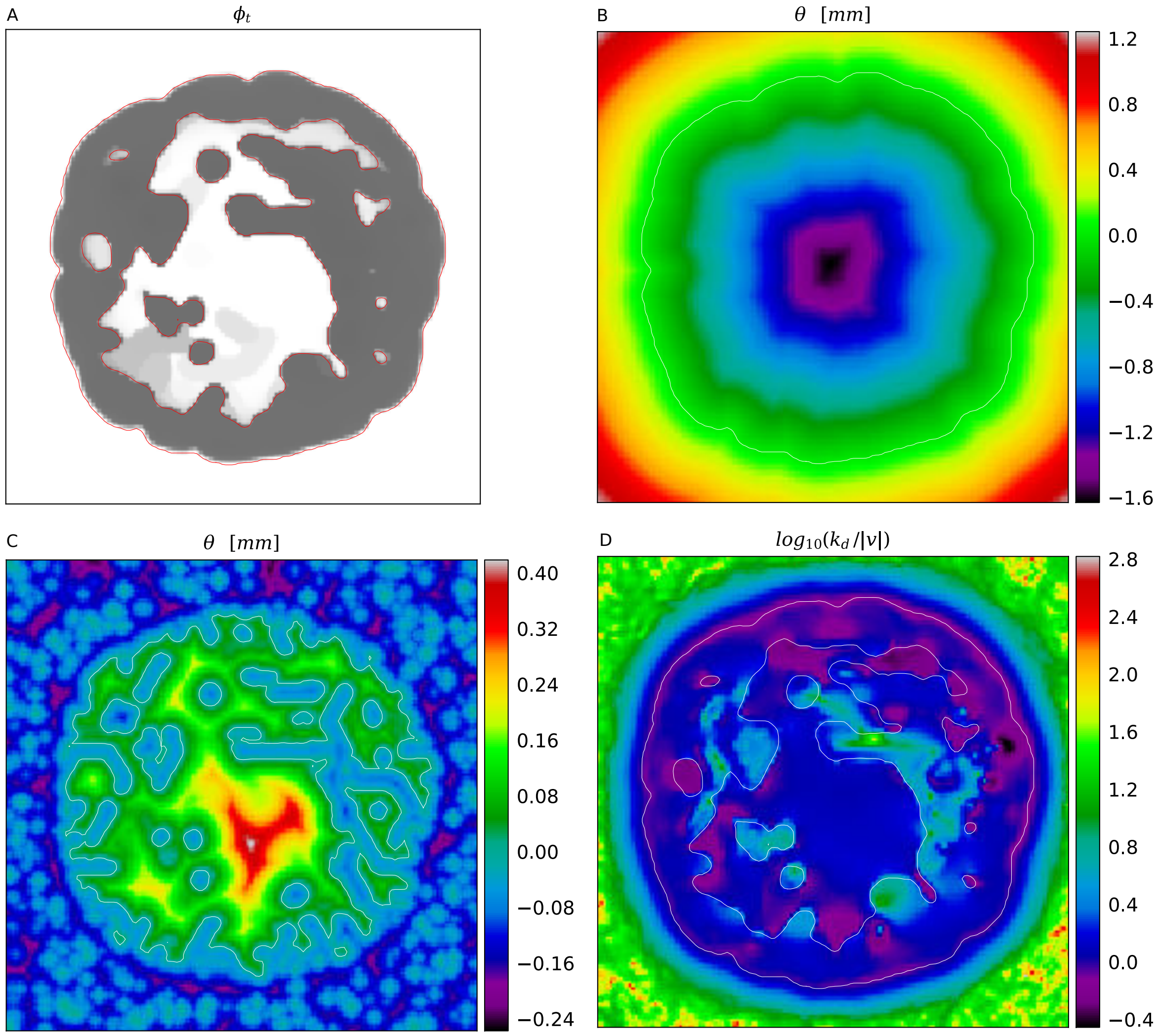

Figure 1: ***M***aps of (A) the viable tumor cell volume fraction, (B) distance from the tumor boundary θ, (C) distance from vessels, and (D) logarithmic plot of the length scale L_{dc} which is defined by requiring that the Peclet number equals one, i.e. $1 = Pe = L_{dc}|v|/k_d$.

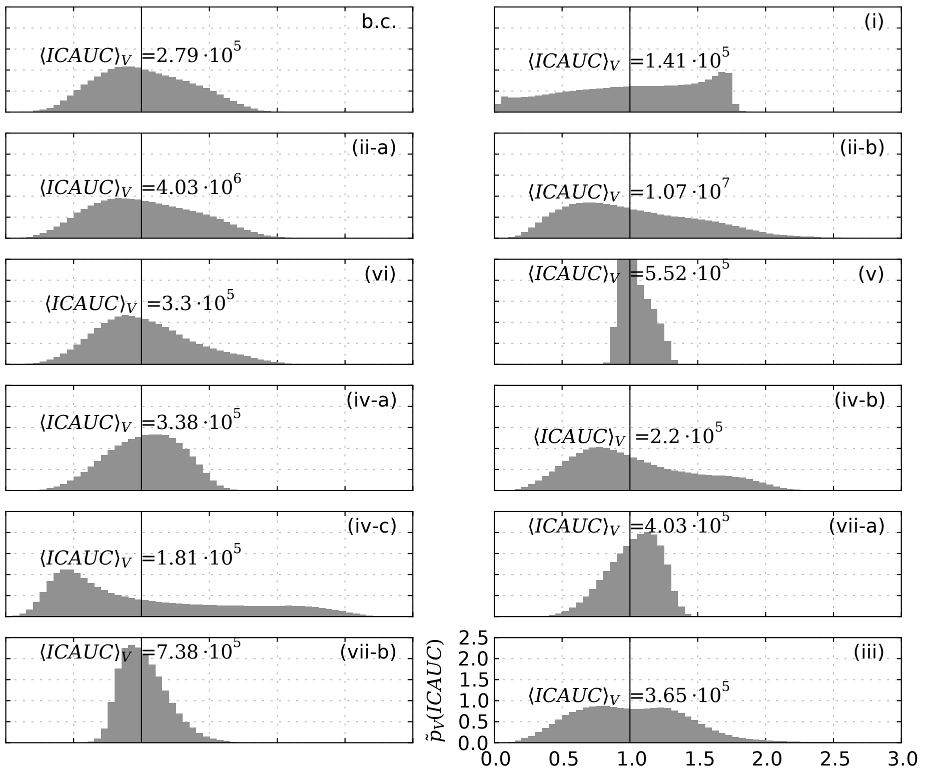

Figure 2: ***C***omparison of the probability density functions (PDFs) of ICAUC over the viable tumor mass (indicated by V) for the various cases. The mean of the distribution is given as text and marked by the vertical line. On the ordinate, the relative deviation from the mean is plotted. The PDFs are computed by histograms summarizing ICMAX and ICAUC values of all runs from the viable tumor region Ω_V.

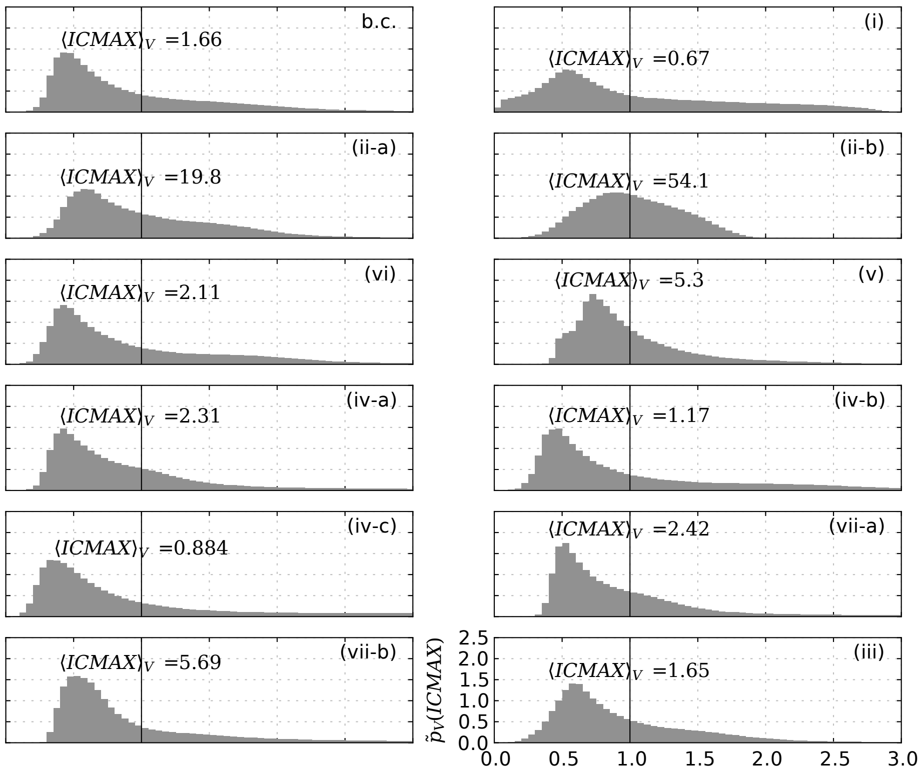

Figure 3: ***C***omparison of the probability density functions of ICMAX over the viable tumor mass (indicated by V) for the various cases. The presentation is analogous to Figure 2.
